# Biomaterial-Mediated Genetic Reprogramming of Merkel Cell Carcinoma and Melanoma Leads to Targeted Cancer Cell Killing *In Vitro* and *In Vivo*

**DOI:** 10.1021/acsbiomaterials.3c00885

**Published:** 2023-10-05

**Authors:** Kathryn M Luly, Jordan J Green, Joel C Sunshine, Stephany Y Tzeng

**Affiliations:** † Department of Biomedical Engineering, Johns Hopkins University, Baltimore, Maryland 21205, United States; ‡ Translational Tissue Engineering Center, Johns Hopkins University School of Medicine, Baltimore, Maryland 21231, United States; § Institute for Nanobiotechnology, Johns Hopkins University, Baltimore, Maryland 21218, United States; ∥ Bloomberg∼Kimmel Institute for Cancer Immunotherapy, Johns Hopkins University School of Medicine, Baltimore, Maryland 21231, United States; ⊥ Sidney Kimmel Comprehensive Cancer Center, Johns Hopkins University School of Medicine, Baltimore, Maryland 21231, United States; # Departments of Neurosurgery, Ophthalmology, and Oncology, Johns Hopkins University School of Medicine, Baltimore, Maryland 21231, United States; ∇ Departments of Materials Science & Engineering and Chemical & Biomolecular Engineering, Johns Hopkins University, Baltimore, Maryland 21218, United States; ○ Departments of Dermatology and Pathology, Johns Hopkins University School of Medicine, Baltimore, Maryland 21287, United States

**Keywords:** nanomaterials, immunotherapy, gene delivery, melanoma, Merkel cell carcinoma

## Abstract

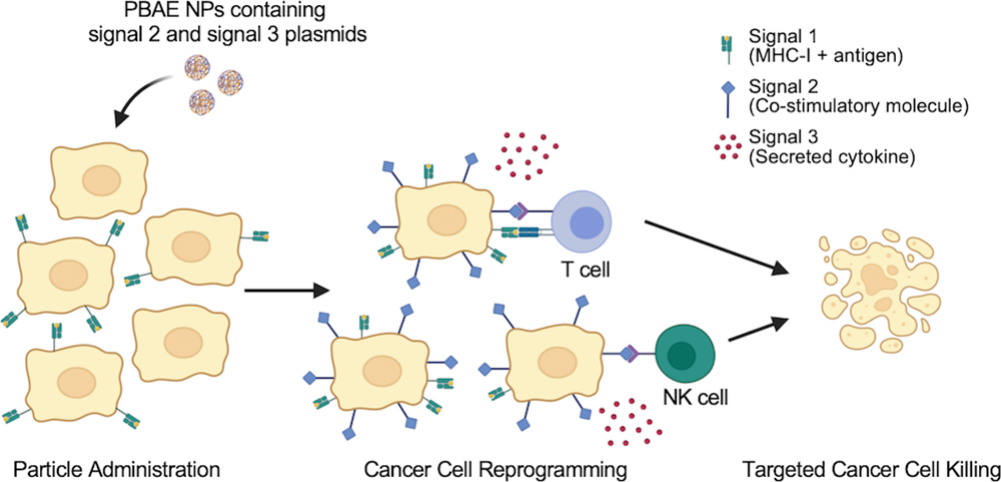

Tumor immunotherapy is a promising anticancer strategy; however, tumor cells may employ resistance mechanisms, including downregulation of major histocompatibility complex (MHC) molecules to avoid immune recognition. Here, we investigate reprogramming nanoparticles (NPs) that deliver immunostimulatory genes to enhance immunotherapy and address defective antigen presentation in skin cancer *in vitro* and *in vivo*. We use a modular poly(beta-amino ester) (PBAE)-based NP to deliver DNA encoding 4-1BBL, IL-12, and IFNγ to reprogram human Merkel cell carcinoma (MCC) cells *in vitro* and mouse melanoma tumors *in vivo* to drive adaptive antitumor immune responses. Optimized NP formulations delivering 4-1BBL/IL-12 or 4-1BBL/IL-12/IFNγ DNA successfully transfect MCC and melanoma cells *in vitro* and *in vivo*, respectively, resulting in IFNγ-driven upregulation of MHC class I and II molecules on cancer cells. These NPs reprogram the tumor immune microenvironment (TIME) and elicit strong T-cell-driven immune responses, leading to cancer cell killing and T-cell proliferation *in vitro* and slowing tumor growth and improving survival rates *in vivo*. Based on expected changes to the tumor immune microenvironment, particularly the importance of IFNγ to the immune response and driving both T-cell function and exhaustion, next-generation NPs codelivering IFNγ were designed. These offered mixed benefits, exchanging improved polyfunctionality for increased T-cell exhaustion and demonstrating higher systemic toxicity *in vivo*. Further profiling of the immune response with these NPs provides insight into T-cell exhaustion and polyfunctionality induced by different formulations, providing a greater understanding of this immunotherapeutic strategy.

## Introduction

Merkel cell carcinoma (MCC) is a rare but aggressive skin cancer with 46% mortality,^1^ while melanomas are more common and responsible for 75% of skin cancer deaths.^2^ Although immune checkpoint inhibitors (ICIs) have revolutionized treatment,^3−5^ resistance often arises, and approximately half of patients do not respond.^2,6^ Immune activation against cancer requires the presentation of tumor antigens in the correct context, a pro-inflammatory tumor immune microenvironment (TIME), and a responsive adaptive immune system. Presentation of peptides by major histocompatibility complexes I and II (MHC-I and -II) is critical for successful anticancer immune responses. Effective antigen presentation by MHC-I, required for immune recognition by CD8+ T cells (“signal 1”),^7,8^ occurs alongside costimulatory signals on the surface of the antigen-presenting cell (“signal 2”) and secreted signals directing cellular fate (“signal 3”). Tumors that respond well to immunotherapy have a “hot” TIME consisting of higher numbers of early activated cytotoxic T cells and low numbers of tumor-associated macrophages.^9^ One resistance mechanism employed by tumors with a “cold” TIME and associated with cancer cells broadly is decreased expression of MHC-I, limiting a cytotoxic cellular response.^10^ Downregulation of MHC-I can result from genetic loss or transcriptional repression,^11^ although restoration of MHC-I expression in *ex vivo* cultures demonstrates that loss of expression is often reversible.^12^

Given the incomplete responses and resistance to ICI therapy, there is a need to develop targeted therapeutics that can potentiate antitumor immune responses. Biodegradable nanoparticles (NPs) can meet these challenges by engineering immune cell function and generating specific immune stimulation against cancer cells. Poly(beta-amino ester)s (PBAEs) can be synthesized from inexpensive commercially available reagents and do not have the same size restrictions on genetic cargo that often limit viral vectors.^13^ The resulting NPs are modular, allowing for replacement and combination of genetic cargo with ease.^14−16^ Previous work has demonstrated that codelivering DNA plasmids encoding immunostimulatory molecules can generate targeted immune responses without prior knowledge of tumor antigens.^17^ Here, we use PBAE-based NPs delivering plasmids encoding signal 2 (4-1BBL) and signal 3 (IL-12) for immune activation against human-patient-derived MCC samples *in vitro* and mouse melanoma *in vivo*. The flexibility afforded by our biomaterial platform allows additional DNA cargoes to be easily tested with this system, and we leverage this to evaluate whether the NPs’ antitumor effect can be improved by the addition of interferon-γ (IFNγ) DNA via interferon-driven modulation of MHC-I expression. We profile subsequent immune responses, evaluating MHC-I modulation and markers of T-cell polyfunctionality and exhaustion *in vivo*, giving insight into the immune mechanisms associated with NP-based TIME reprogramming.

## Materials and Methods

### Polymer Synthesis

1,4-Butanediol diacrylate (B4, Alfa Aesar), 1,5-pentanediol diacrylate (B5, SantaCruz Biotechnology), 3-amino-1-propanol (S3, Alfa Aesar), 4-amino-1-butanol (S4, Fisher Scientific), 5-amino-1-pentanol (S5, Alfa Aesar), 2-(3-aminopropylamino)ethanol (E6, Sigma-Aldrich), 1-(3-aminopropyl)-4-methylpiperazine (E7, Alfa Aesar), 4,7,10-trioxa-1,13-tridecanediamine (E27, Sigma-Aldrich), N,N-dimethyldipropylenetriamine (E49, Sigma-Aldrich), and pentaethylenehexamine (E60, Santa Cruz) were purchased and stored according to the manufacturer’s instructions. As shown in Figure S1, acrylate backbone (B) and amine side chain (S) monomers were mixed at a 1.1:1 molar ratio and stirred neat at 85 °C for 24 h. The resulting acrylate-terminated B–S polymers were dissolved in anhydrous tetrahydrofuran (THF) at 200 mg/mL, combined with an end-capping monomer, and allowed to react at 25 °C for 2 h. End-capped polymers were precipitated in diethyl ether, washed twice, dried under vacuum for 2–3 days, then dissolved in anhydrous dimethyl sulfoxide (DMSO) and stored at −20 °C on desiccant.

### Polymer Characterization

Ether-precipitated 4,7,10-trioxa-1,13-tridecanediamine end-modified poly(1,4-butanediol diacrylate-*co*-5-amino-1-pentanol) (referred to as B4–S5–E27 or 4–5–27) was dissolved at 10 mg/mL in THF, and the molecular weight was determined via gel permeation chromatography (GPC) relative to polystyrene standards using a refractive index detector (Agilent). Ether-precipitated 4–5–27 was also dissolved at 10 mg/mL in DMSO-*d*_6_ and characterized using ^1^H nuclear magnetic resonance (NMR) on a Bruker 500 MHz NMR. The structure of 4–5–27 was confirmed using TopSpin4.1.4 (Bruker).

### Nanoparticle Characterization

pEGFP-N1 plasmid (Clontech, amplified and purified by Elim Biopharmaceuticals) and 4–5–27 were each dissolved in 25 mM sodium acetate buffer (pH 5) and combined at a 1:1 ratio (v/v) at varying mass ratios with vigorous mixing. NPs self-assembled within minutes at room temperature and were then diluted 5-fold for dynamic light scattering (DLS) and 100-fold for nanoparticle-tracking analysis (NTA) in 1× phosphate-buffered saline (PBS). NP size, measured by hydrodynamic radius, was determined by DLS using a Malvern Zetasizer Pro (Malvern Panalytical) and by NTA using a NanoSight NS300 (Malvern Panalytical). NPs were also diluted 5-fold in a solution of 0.1× PBS, and surface charge, measured as zeta potential, was determined also using a Malvern Zetasizer Pro. For each assay, *n* = 3 measurements were taken on individually prepared replicates.

### Cell Culture

MCC13, UISO, and MCC26 cells were provided by Isaac Brownell (National Institutes of Health). Cells were cultured in RPMI 1640 (Gibco, Thermo Scientific) with 10% FBS (Sigma) and 1% penicillin–streptomycin (Gibco, Thermo Scientific) at 37 °C, 5% CO_2_, and a humidified environment. For all *in vitro* assays, 5000 cells/well were plated well in 96-well plates and allowed to adhere overnight before transfection.

### *In Vitro* PBAE Transfection and Screening

All *in vitro* transfection and screening results represent *n* = 4 biological replicates. PBAE polymer and plasmid DNA were diluted separately in 25 mM sodium acetate buffer (pH 5) and combined (1:1 v/v ratio and 30–120 w/w ratio of polymer to DNA) at room temperature for self-assembly within minutes. The resulting NPs were administered to cells at 300–600 ng of DNA/well and incubated for 2 h at 37 °C, followed by a media change. The pEGFP-N1 (Elim Biopharmaceuticals) plasmid was used for polymer screening. GFP expression in MCC13, UISO, and MCC26 was assessed 48 h following transfection via an Attune NxT flow cytometer (ThermoFisher). Toxicity was assessed via MTS using CellTiter 96 AQ_ueous_ One Solution Cell Proliferation Assay (Promega) 24 h following transfection according to the manufacturer’s instructions or via relative cell counts measured by flow cytometry 48 h following transfection.

To assess 4-1BBL (pUNO1-h41BBL, Invivogen) and IL-12 [pUNO1-hIL12(p40p35), Invivogen] plasmid delivery, cells were transfected as described above with 450 ng of DNA/well in 96-well plates (3.75 μg/mL final DNA concentration) and varying ratios of 4-1BBL:IL-12 plasmids or control plasmid (GFP). Cells and media were collected 48 h following transfection, stained for 4-1BBL (Biolegend, clone 5F4, cat. no. 311506, 1:20 dilution), and assessed via flow cytometry. IL-12 secretion into the culture medium was assessed via ELISA (Biolegend catalog no. 431704).

For NPs delivering plasmids for 4-1BBL, IL-12, and IFNγ (pUNO1-hIFNγ, Invivogen), plasmids were mixed during formulation (see Figure S5C), and IFNγ expression was confirmed via ELISA (Biolegend cat. no 430104). To assess MHC-I induced by IFNγ plasmid expression compared to that induced by spiked-in recombinant IFNγ protein, MCC13 and UISO were plated and treated with control (GFP) NPs, 4-1BBL/IL-12/IFNγ NPs, or recombinant IFNγ (Biolegend, cat. no. 570204) at a final concentration of 1, 5, 10, 25, or 50 ng/mL. Cells were harvested 1, 2, or 3 days later, fixed for 30 min in 4% formaldehyde, and stored at 4 °C. When all time points were collected, cells were stained for MHC-I (APC-HLA-A,B,C, Biolegend, clone W6/32, cat. No. 311410), and MHC-I mean fluorescence intensity (MFI) was determined via flow cytometry.

### *In Vitro* MHC-I Immunofluorescence

Cells were plated and transfected as above. Four days later, cells were fixed for 30 min in 4% neutral buffered formaldehyde, followed by blocking in 5% milk in PBS-T (0.05% Tween-20 in PBS) for 1 h. Cells were stained for MHC-I (APC-HLA-A,B,C, Biolegend, clone W6/32, cat. No. 311410, 1:20 dilution in 5% milk in PBS-T) for 2 h at room temperature. Cells were washed three times with PBS-T, then stained in PBS-T with 10 μg/mL Hoechst 33342 (Life Technologies H3570) for 30 min at 37 °C. Cells were imaged on a Zeiss Axio Observer fluorescence microscope (Zeiss).

### CD8+ and PBMC Coculture Assays

All coculture assay results represent *n* = 4 biological replicates. Human CD8+ T cells were obtained from Cellero (MA, USA; formerly Astarte). Peripheral blood mononuclear cells (PBMCs) were isolated from a Leukopak donation from a de-identified donor (Hemapheresis and Transfusion Center, Johns Hopkins Hospital). For coculture assays, MCC cells were transfected as above [GFP used as control plasmid in CD8+ coculture assay (Figure 3) and PBMC optimization (Figure S5), fLuc used as control plasmid in PBMC coculture assays (Figures 4, S6)]. The next day, 5000 or 50,000 CD8+ T cells or 15,000 or 100,000 PBMCs were added. For the CD8+ T-cell coculture, media was supplemented with 20 U/mL IL-2 (PeproTech Inc.) when the T cells were added and replenished on days 3 and 5. For PBMC coculture, media was not supplemented with IL-2 and was replaced on days 3 and 5. IFNγ ELISA (Biolegend, cat. no. 430104) on the media and flow cytometry on the cells were performed on days 3 and 7 (Tables S1 and S2).

### NP Formation for *In Vivo* Transfection

NPs were formed using PBAE and 10 μg of plasmid at a polymer-to-DNA mass ratio of 30 w/w in sodium acetate buffer (pH 7) per 50 μL injection. Up to three plasmids were coformulated and delivered in NPs: firefly luciferase (fLuc, pcDNA3-fLuc, Elim Biopharmaceuticals), 4-1BBL (pUNO1-m41BBL, Invivogen), IL-12 (mIL12-N1, Addgene plasmid #123139^17^), and IFNγ (pUNO1-mIFNγ, Invivogen). When fewer than three therapeutic plasmids were delivered, the remaining mass of the plasmid cargo was made up of fLuc DNA. NPs were frozen at −80 °C and thawed immediately before use.

### B16F10 *In Vivo* Model

All animal work was conducted within the guidelines of the Johns Hopkins Animal Care and Use Committee under approved protocol number MO18M388/MO21M384. Eight-week-old C57BL/6J female mice (Jackson Laboratory) were anesthetized with isoflurane, their flanks were shaved, and 3 × 10^5^ B16F10 cells in unsupplemented RPMI media were injected subcutaneously (50 μL volume). To assess *in vivo* transfection, particles encapsulating fLuc plasmid DNA were formed as above, and 50 μL was injected intratumorally in *n* = 3 animals. Twenty-four hours following NP injection, 150 mg/kg d-luciferin (Cayman Chemicals) was administered subcutaneously, and mice were imaged on an In Vivo Imaging System (IVIS) Spectrum (PerkinElmer) 7 min later.

To assess therapeutic NPs, tumors were implanted as described above. The day before treatment, tumors were measured, and mice were assigned to groups with an equal initial tumor size. After 9, 11, 16, and 18 days, mice were anesthetized, and 50 μL of NPs was injected into the tumors. Where indicated, 200 μg of anti-PD-1 antibody (clone RMP1–14, BioXCell) was also administered intraperitoneally on the same days. For long-term studies, the tumor area, defined as tumor length × tumor width, was measured every other day by caliper in *n* = 8 mice by a researcher blinded to experimental groups. Animals were sacrificed once tumors reached 200 mm^2^ or earlier if moribund.

To analyze transfected tumors, therapeutic NPs were administered as described above to *n* = 4 or 5 melanoma-bearing C57BL/6J female mice. Mice were sacrificed 20 days after tumor injection. A portion of the tumor with overlying skin was fixed in 10% neutral buffered formalin. The remaining tumor was excised. A small portion was saved and stored at −80 °C for qRT-PCR, and the rest was minced and digested in 2 mg/mL Collagenase D (Roche) at 37 °C for 1 h. Samples were then passed through a 70-μm cell strainer, rinsed with PBS, and pelleted at 300 × rcf for 5 min. Pellets were treated with ACK lysis buffer (Quality Biological) for 1 min, diluted with 20 mL of PBS, and filtered through a 100-μm cell strainer. Cells were pelleted again and then stained for flow cytometry analysis in 1× PBS with 2% FBS (Tables S3 and S4).

For intracellular cytokine staining (ICS), cells were processed as above and cultured in RPMI supplemented with 10% FBS (Sigma), 1% penicillin–streptomycin (Gibco, Thermo Scientific), 0.1% 2-mercaptoethanol (v/v), and 1× cell stimulation cocktail plus protein transport inhibitors (eBioscience, Invitrogen) for 3 h. Stimulated cells were then fixed and permeabilized using a BD Cytofix/Cytoperm kit (BD Biosciences) according to the manufacturer’s instructions and then stained for flow cytometry analysis (Table S5).

### Histology

Fixed tumors (*n* = 4 per group) were sectioned and stained by standard hematoxylin and eosin (H&E) and for CD8 by the Johns Hopkins Oncology Tissue Services core. Blinded, semiquantitative scoring of H&E- and CD8-stained slides was performed by a board-certified dermatopathologist (JCS). The degree of inflammation and CD8 infiltration was scored as defined: 0 = none, 1 = mild, 2 = moderate, 3 = strong.

### Analysis via qPCR

RNA was isolated from samples (*n* = 4 per group) using the Direct-zol RNA MiniPrep kit (Zymo Research). cDNA was synthesized using an iScript cDNA synthesis kit (Bio-Rad). qPCR was conducted using Power SYBR Green Master Mix (Thermo Fisher Scientific) on a StepOnePlus Real-Time PCR System (Thermo Fisher Scientific). Differences in expression were assessed by delta delta *C*_T_.

### Statistical Methods

Statistical tests were performed using GraphPad Prism 8 Software. Groups were analyzed via one-way ANOVA with Dunnett’s post-tests to compare to a control group. For PBMC coculture with MCC cells cotransfected with IFNγ, significance was assessed using one-way ANOVA comparing each group to a matched control with Bonferroni correction for multiple comparisons. Differences in tumor sizes were assessed via two-way ANOVA with Dunnett’s post-test comparing all groups to control. Significant differences in survival were assessed via the Log-Rank (Mantel-Cox) test with Bonferroni correction for multiple comparisons. Adjusted *p*-values of *p* < 0.05 were considered significant and denoted: *, *p* < 0.05; **, *p* < 0.01; ***, *p* < 0.001.

## Results

### NP Screening and Optimization in Human MCC Cell Lines

**Figure 1:**
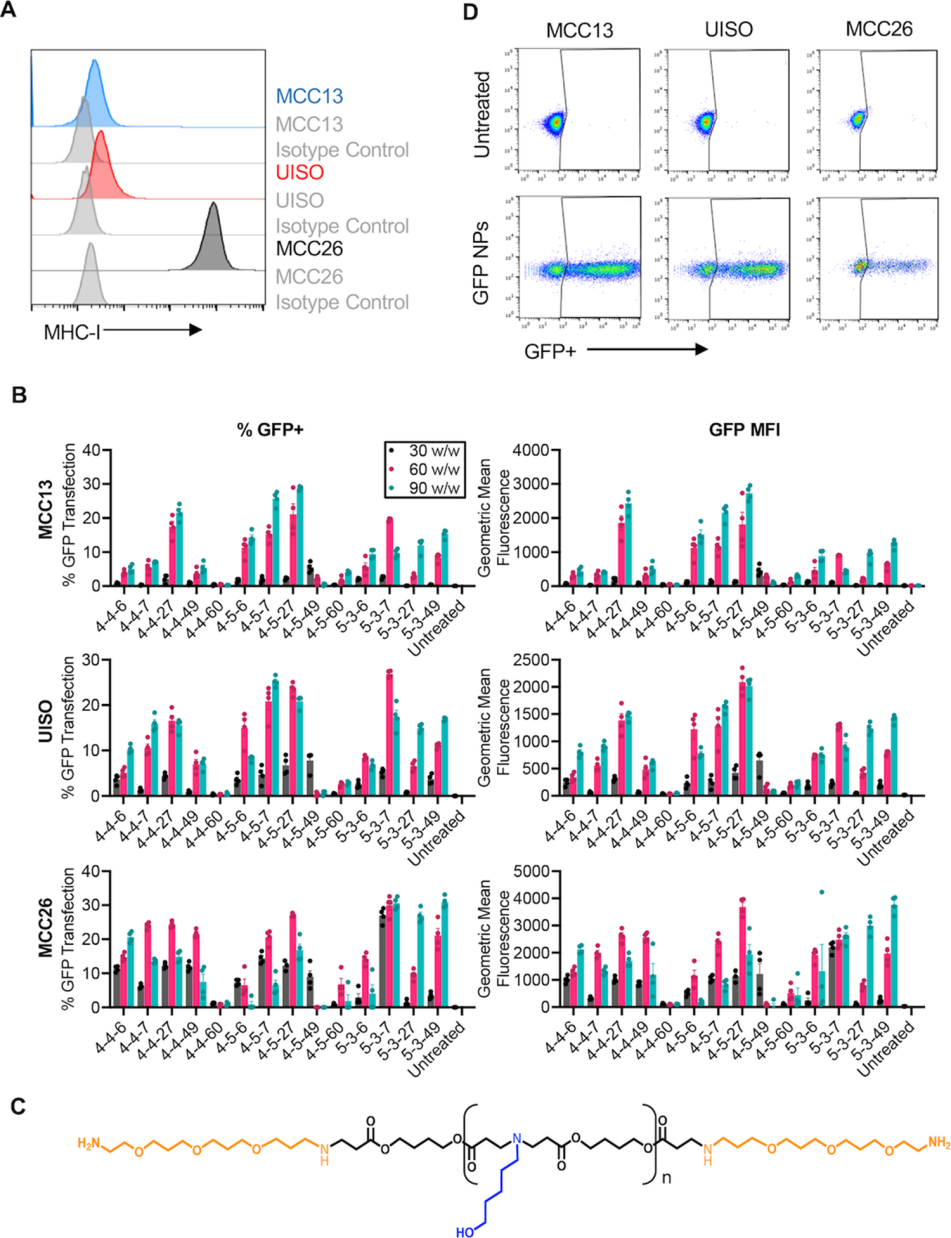
PBAE screening in three human MCC cell lines. (A) Three human MCC cell lines, MCC13, UISO, and MCC26, have varying baseline expression of MHC-I. (B) PBAE NPs delivered 600 ng of GFP DNA at 30, 60, and 90 w/w to MCC13, UISO, and MCC26. Percent GFP transfection and GFP geometric mean fluorescence were assessed via flow cytometry 48 h following transfection. (C) Structure of PBAE 4–5–27. (D) 4–5–27 NPs delivering GFP DNA led to robust transfection of MCC13, UISO, and MCC26 cells *in vitro*.

**Figure 2:**
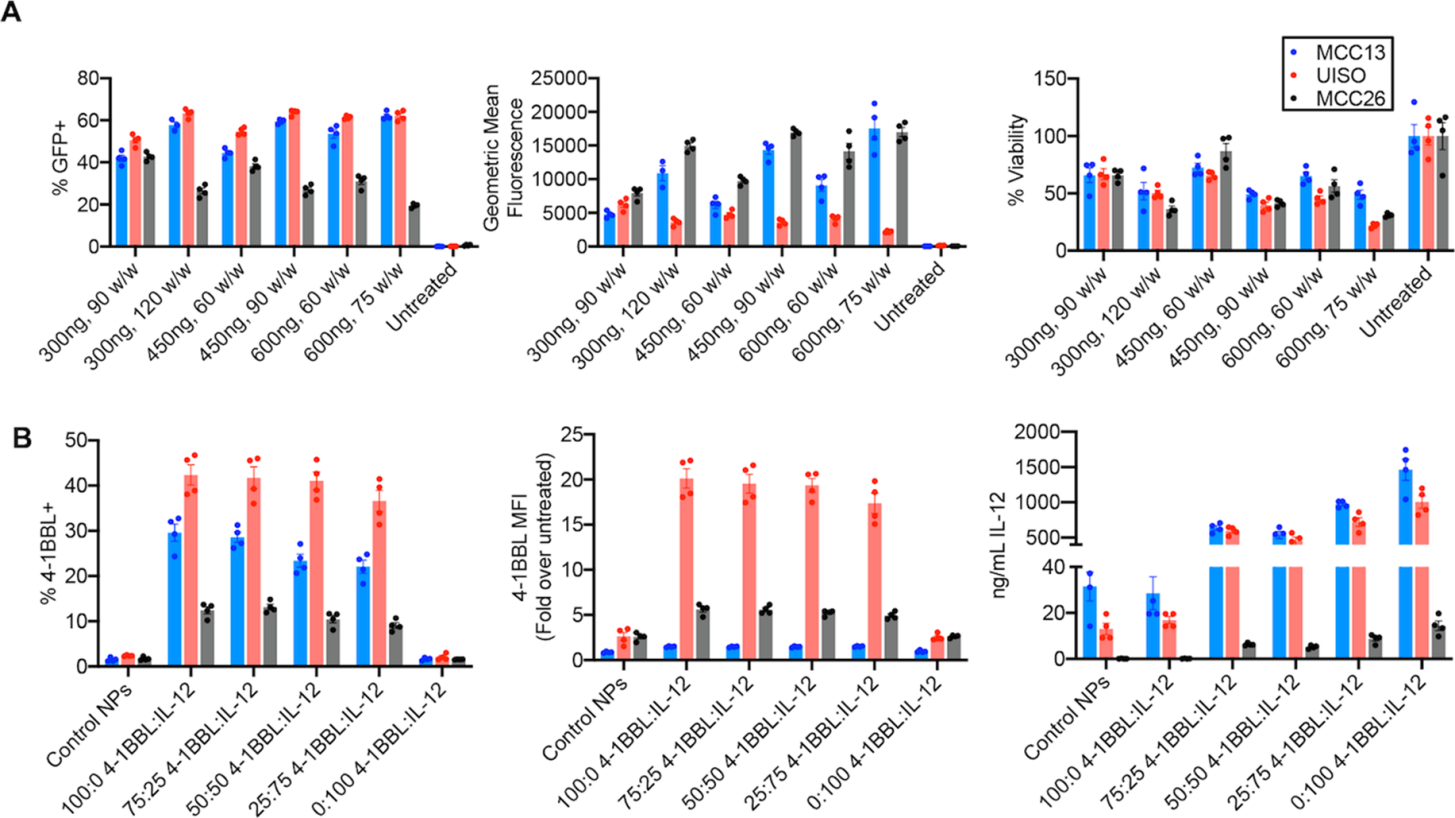
Optimization of 4–5–27 NP-mediated DNA delivery to MCC cells. (A) NPs delivering 300 ng of GFP DNA at 90 or 120 w/w, 450 ng at 60 or 90 w/w, or 600 ng at 60 or 75 w/w were administered to MCC cells. Percent GFP positive cells, GFP MFI, and cellular viability were assessed via flow cytometry 48 h following transfection. (B) NPs delivering 450 ng total plasmid DNA were formed with various ratios of 4-1BBL and IL-12 plasmids or with control plasmid (GFP). Percent 4-1BBL transfection and 4-1BBL MFI were assessed via flow cytometry 48 h following transfection. IL-12 secretion in media was assessed via ELISA 48 h following transfection.

A library of 14 PBAEs of varying structure and properties were synthesized (Figure S1). NPs encapsulating a GFP reporter gene were formulated at several DNA doses and polymer:DNA w/w ratios. NP formulations were assessed for robust GFP expression in three human Merkel cell polyomavirus-negative MCC cell lines, MCC13, UISO, and MCC26, which have varying levels of baseline MHC-I: MCC13 and UISO have low baseline MHC-I, whereas MCC26 has high baseline MHC-I (Figure 1A). Successful NP delivery was measured as the percentage of GFP+ cells and geometric mean GFP fluorescence 48 h following transfection (Figure 1B), as well as lack of nonspecific toxicity (Figure S2). Based on the screening results, 4,7,10-trioxa-1,13-tridecanediamine end-modified poly(1,4-butanediol diacrylate-*co*-5-amino-1-pentanol) (henceforth “PBAE 4–5–27,” Figure 1C, further characterized in Figure S3A,B) was chosen for subsequent experiments due to its high transfection efficacy and low toxicity across all three cell lines when delivering 450 ng of DNA at 60 w/w ratio of polymer to DNA (Figures 1D and 2A).

As our strategy relies on combined delivery of two therapeutic genes, IL-12 and 4-1BBL, we then tested different ratios of 4-1BBL and IL-12 plasmids in the MCC cell lines. 4-1BBL expression, measured as percent 4-1BBL+ cells and geometric mean fluorescence, was the highest in UISO cells and the lowest in MCC26 cells, although expression within each cell line remained relatively stable as the ratio of 4-1BBL plasmid in the NPs decreased (Figure 2B). IL-12 secretion into media showed a dose response, with more IL-12 secreted as the proportion of IL-12 plasmid in the NPs increased (Figure 2B). Thus, a final NP formulation of 25% 4-1BBL plasmid and 75% IL-12 plasmid, with 450 ng of total DNA at 60 w/w, was chosen for subsequent experiments, allowing robust expression of both 4-1BBL and IL-12 in all three MCC cell lines (Figure 2B).

### Coculture of Transfected Human MCC Cells with Human CD8+ T Cells

**Figure 3:**
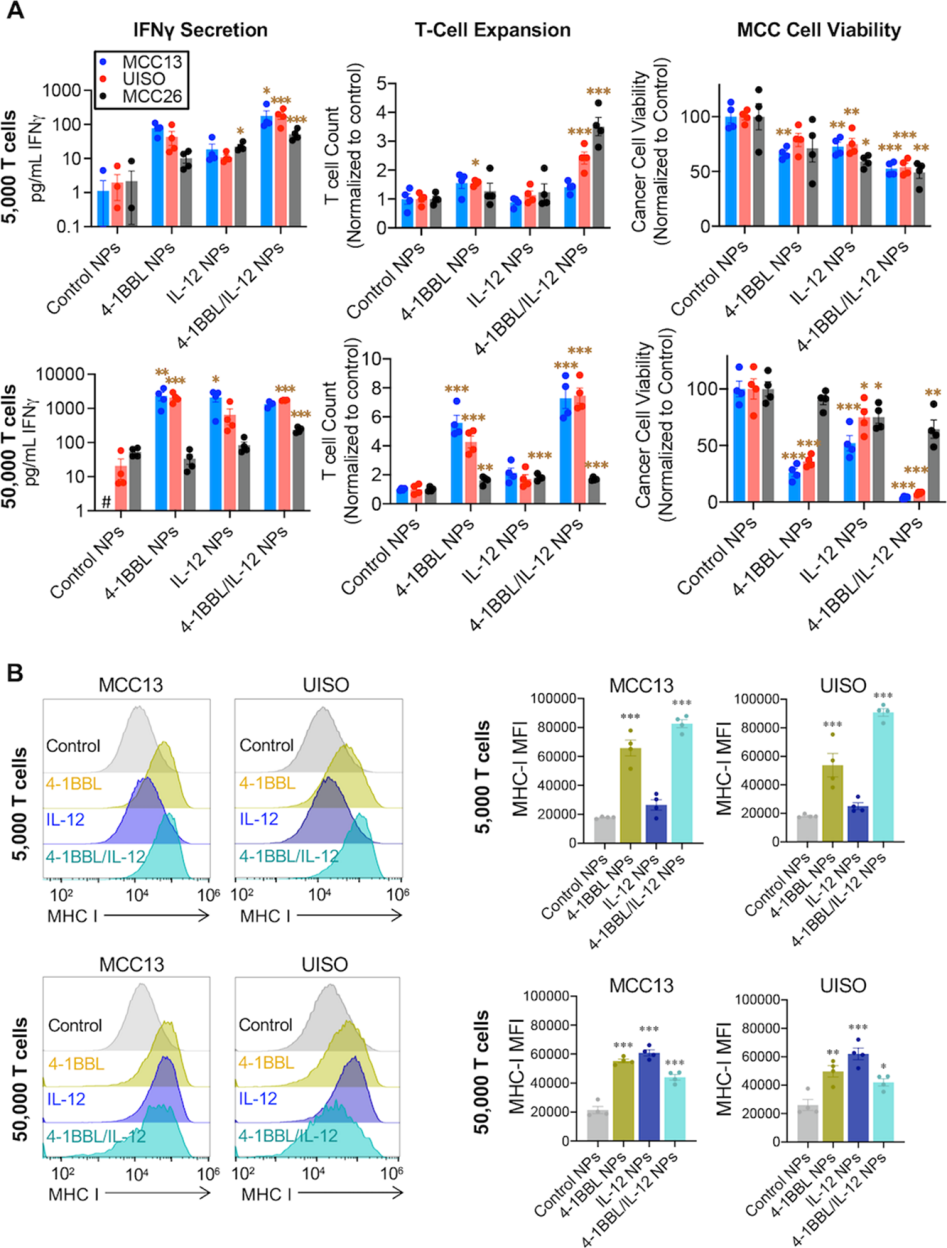
Coculture of transfected MCC cell lines with human CD8+ T cells. For coculture studies, MCC cells were transfected with NPs delivering control plasmid (GFP), plasmids for 4-1BBL, IL-12, or both. 5000 or 50,000 human CD8+ T cells were added 24 h following transfection, and media was supplemented with 20 U/mL IL-2. Significance in comparison to control, via one-way ANOVA and Dunnett’s post-test, is represented. Error bars are SEM. # denotes conditions below the limit of detection. Significance is denoted: *, *p* < 0.05; **, *p* < 0.01; ***, *p* < 0.001. (A) IFNγ secretion was assessed via ELISA, and CD8+ T-cell expansion and MCC cell killing were assessed via flow cytometry 7 days following the addition of CD8+ T cells. (B) MHC-I induction, represented as histograms (left) and MFI (right) in cell lines with low baseline MHC, was assessed via flow cytometry 7 days following the addition of CD8+ T cells.

To test the ability of 4-1BBL/IL-12 NPs to reprogram MCC cells and activate antitumor immune responses, we transfected MCC cells with 4-1BBL/IL-12 NPs and cocultured them with human CD8+ T cells the following day. IFNγ secretion, T-cell expansion, and cancer cell viability were assessed on day 7 by ELISA and flow cytometry (Figures 3A, S4A,B). We measured significantly higher IFNγ secretion after administration of 4-1BBL/IL-12 NPs in all three cell lines cocultured with 5000 CD8+ T cells when compared to control NPs, as well as increased IFNγ secretion with therapeutic NPs in MCC13 and UISO with 50,000 CD8+ T cells (Figure 3A). Significant T-cell expansion was observed in all three cell lines with 4-1BBL and 4-1BBL/IL-12 NPs with 50,000 CD8+ T cells (Figure 3A). Additionally, there were significantly lower cancer cell counts in all cell lines with 4-1BBL/IL-12 NPs compared to control NPs with 5000 or 50,000 CD8+ T cells (Figure 3A). MHC-I expression increased in MCC13 and UISO, the lines with low pretreatment MHC I expression, when MCC cells transfected with 4-1BBL NPs or 4-1BBL/IL-12 NPs were cocultured with 5000 CD8+ T cells, and when MCC cells were transfected with any therapeutic NPs and cocultured with 50,000 CD8+ T cells (Figure 3B).

### Coculture of Transfected Human MCC Cells With Human PBMCs

**Figure 4:**
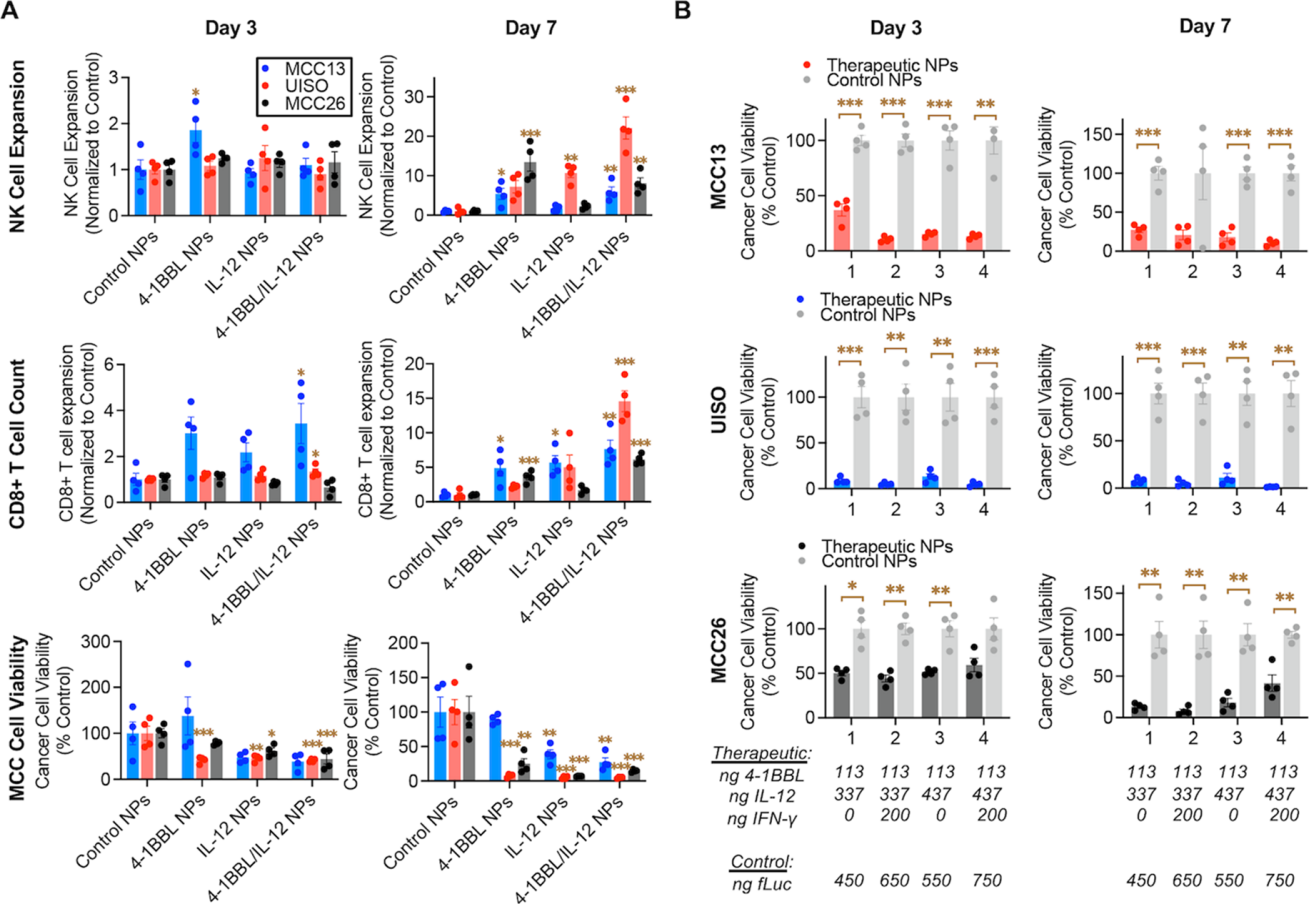
Coculture of human MCC cells with human PBMCs. MCC cells were transfected with NPs delivering control plasmid (fLuc) or plasmids for 4-1BBL, IL-12, and IFNγ (in B, nanogram amounts of each plasmid are indicated). Human PBMCs (100,000/well in A, 15,000/well in B) were added 24 h following transfection. NK-cell expansion, T-cell expansion, and MCC cell killing were assessed via flow cytometry 3 and 7 days following the addition of PBMCs. Error bars are SEM. In A, significance compared to control, via one-way ANOVA and Dunnett’s post-test, for each cell line is represented. In B, significance, via one-way ANOVA comparing each group to its matched control with Bonferroni correction for multiple comparisons, for each cell line is represented. Significance is denoted: *, *p* < 0.05; **, *p* < 0.01; ***, *p* < 0.001.

Because CD8+ T cells are not isolated in the *in vivo* TIME, we evaluated the reprogramming NPs in a coculture model of MCC cells with human PBMCs to evaluate the impact in the context of other immune cells. MCC cells were transfected with control, 4-1BBL, IL-12, or 4-1BBL/IL-12 NPs, and 10^5^ human PBMCs were added per well the following day. On days 3 and 7, immune cells and cancer cells were stained for analysis via flow cytometry (Figure S4C). Although only 4-1BBL-transfected MCC13 cells induced natural killer (NK)-cell expansion at day 3, by day 7, the NK cell count was 6.0-, 22.1-, and 8.1-fold higher with 4-1BBL/IL-12 NP treatment relative to control NPs in MCC13, UISO, and MCC26 coculture models, respectively (Figure 4A, top). The CD8+ T-cell count increased slightly in MCC13 and UISO with 4-1BBL/IL-12 NPs relative to the control at day 3 and increased 7.6-, 14.6-, and 6.1-fold at day 7 relative to control particles in MCC13, UISO, and MCC26, respectively (Figure 4A, middle). Importantly, combination NPs led to significant (*p* < 0.01) cell killing in all three cell lines, with 27± 6%, 5 ± 1%, and 15 ± 1% viability in MCC13, UISO, and MCC26 cells on day 7, respectively (Figure 4A bottom).

To further improve this coculture model, we next optimized the PBMC seeding density to minimize treatment-independent cell killing and allow for clearer observation of treatment-specific effects. We transfected MCC13 with control GFP NPs or 4-1BBL/IL-12 NPs, added a range of PBMCs from 0 to 10^5^ cells/well the following day, and assessed MCC13 cell count on days 3 and 7 (Figure S5A). To further refine the appropriate seeding density across additional MCC cell lines, 0–2.5 × 10^4^ PBMCs/well were added to transfected MCC13, UISO, and MCC26 cells. MCC cell count was assessed on day 7 by comparing GFP- and 4-1BBL/IL-12-transfected conditions to untreated cells. The best viability window between GFP and 4-1BBL/IL-12 NPs was found with 1.5 × 10^4^ PBMCs, with minimal treatment-independent cell killing as indicated by the window between the viability of untreated and GFP-transfected cells (Figure S5B). Interestingly, there was substantial MCC26 cell killing by immune cells at this seeding density even after transfection with only GFP. This is likely due to the higher baseline expression of MHC-I in this cell line, allowing increased antigen presentation and therefore better target access, further supporting our hypothesis that MHC-I expression on cells is crucial for an effective immune response against MCC; however, after optimization, a difference between the GFP and 4-1BBL/IL-12 group was still measurable in MCC26 cells at this condition.

Given the known role of IFNγ in promoting MHC expression^18,19^ and the correlation seen in the early coculture studies between IFNγ secretion and MCC killing efficacy (Figure 3A), we tested whether adding a plasmid expressing IFNγ to the NPs further promoted antitumor responses. MCC13 cells were plated and transfected with 4-1BBL/IL-12 NPs or 4-1BBL/IL-12/IFNγ NPs with various amounts of each plasmid. High IFNγ secretion was measured with 200 ng of IFNγ plasmid per well (Figure S5C). We also tested the effect of further increasing IL-12 plasmid delivery by transfecting MCC cell lines with these updated formulations of 4-1BBL/IL-12 NPs with or without IFNγ and coculturing them with 1.5 × 10^4^ PBMCs. Significant NK-cell expansion was seen on day 7 with all formulations tested in MCC13 and UISO, with certain formulations also causing significant CD8+ T-cell expansion (Figure S6) and significantly higher MHC-I and MHC-II expression on days 3 and 7 (Figure S6). Importantly, these results were accompanied by significant cancer cell killing with nearly all NP formulations across all cell lines (Figure 4B).

### MHC Modulation with IFNγ *In Vitro*

**Figure 5:**
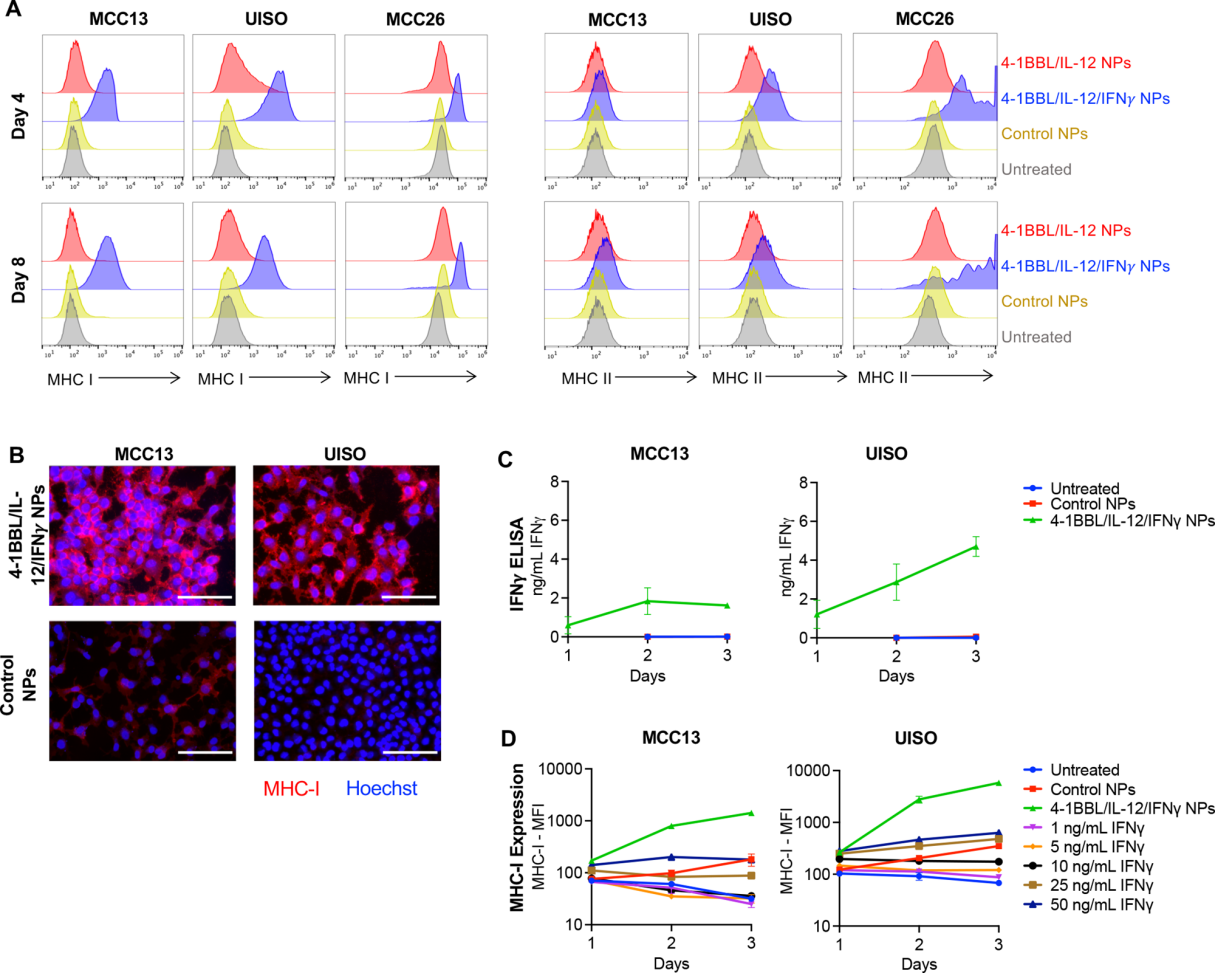
MHC-I and MHC-II induction with reprogramming NPs *in vitro*. (A) MHC-I and MHC-II expression in MCC cells 4 and 8 days following nanoparticle administration (fLuc as a control plasmid). Histograms represent *n* = 4 replicates for each condition. (B) Fluorescence microscopy confirms increased MHC-I expression following administration of signal 2/3 particles compared to control (GFP) particles. (C) MCC13 and UISO were transfected with control (GFP) NPs or 4-1BBL/IL-12/IFNγ NPs. Secreted IFNγ was assessed via ELISA on days 1–3. (D) MCC13 and UISO were transfected with control (GFP) NPs or 4-1BBL/IL-12/IFNγ NPs, or recombinant IFNγ was spiked into media. Cells were stained for MHC-I on days 1–3 for flow cytometry analysis.

To understand how these reprogramming NPs modulate MHC-I, we first assessed MHC-I induction in MCC13, UISO, and MCC26 cells after transfection with 4-1BBL/IL-12 NPs or 4-1BBL/IL-12/IFNγ NPs. MHC-I increased slightly with 4-1BBL/IL-12 NPs in certain cell lines, but both MHC-I and MHC-II showed a marked response in all cell lines after the administration of 4-1BBL/IL-12/IFNγ NPs (Figure 5A). Fluorescence microscopy confirmed this: MCC13 and UISO cells, which have low baseline MHC-I expression, showed a clear increase in MHC-I expression after transfection with 4-1BBL/IL-12/IFNγ NPs compared with cells transfected with control NPs (Figure 5B). We next assessed IFNγ secretion into the medium 1–3 days following administration of 4-1BBL/IL-12/IFNγ NPs and verified that they induce IFNγ secretion in both of the cell lines with low baseline MHC-I expression (Figure 5C). One potential advantage of local autocrine delivery of IFNγ to tumor cells is stronger MHC-I induction compared to bulk dosing of IFNγ in the surrounding environment; therefore, to assess the effect of local delivery of IFNγ on MHC-I induction, we compared 4-1BBL/IL-12/IFNγ NPs to conditions with up to 10-fold higher concentration of soluble recombinant IFNγ than the maximum amount produced by the 4-1BBL/IL-12/IFNγ NPs. 4-1BBL/IL-12/IFNγ NPs generated superior MHC-I induction compared to even the highest concentration of added IFNγ (Figure 5D), demonstrating the advantage of local IFNγ production and giving better insight into the early kinetics of immune modulation with our reprogramming NPs.

### Tumor Size and Survival Analysis of Reprogrammed Mouse Melanoma Tumors

**Figure 6:**
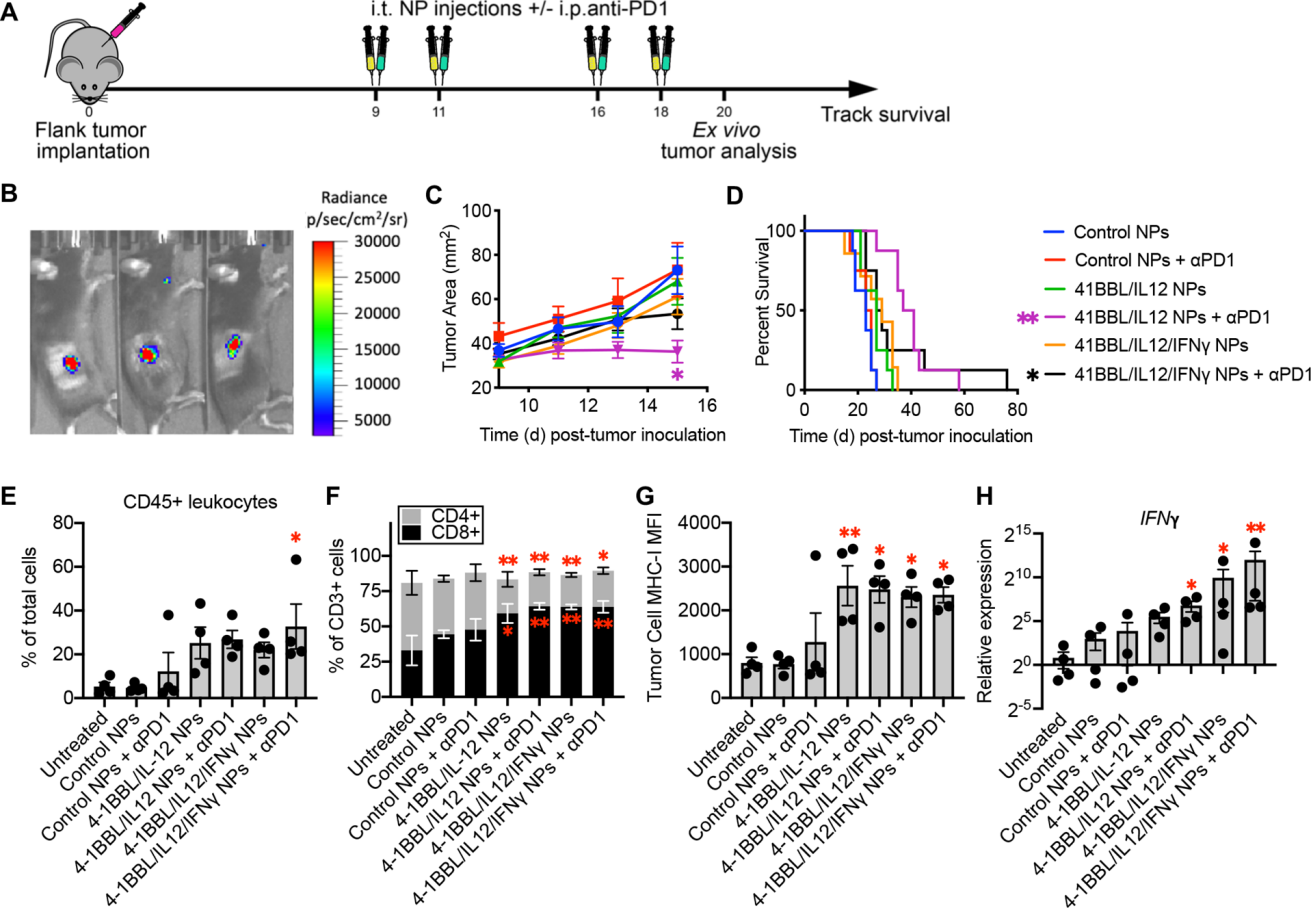
Reprogramming mouse melanoma *in vivo*. (A) Schematic describing *in vivo* studies. B16F10 flank tumors were implanted (*n* = 8 per group in B–C, *n* = 4 per group in D–G), then animals received intratumoral injections of NPs (fLuc used as control) with or without intraperitoneal anti-PD1 on days 9, 11, 16, and 18. Animals were either euthanized and tumors were excised for *ex vivo* analysis on day 20, or tracked for survival. (B) PBAE NP delivery of fLuc DNA demonstrated efficient transfection in B16F10 mouse melanoma tumors. (C) Tumor surface area was measured every other day. On day 15, tumors treated with 4-1BBL/IL12 NPs and anti-PD1 were significantly smaller than tumors treated with control (fLuc) NPs (adjusted *p*-value = 0.0435). Significance assessed via two-way ANOVA with Dunnett’s post-test comparing all groups to control. (D) Animals were sacrificed when tumors reached 200 mm^2^ or for moribund states, and survival was tracked. Significance was assessed via the Log-Rank (Mantel-Cox) test with Bonferroni correction for multiple comparisons. (E) Immune cell infiltration into tumors measured by flow. (F) CD4+ and CD8+ T-cell infiltration measured by flow. (G) Tumor cell MHC-I expression measured by flow. (H) IFNγ expression measured via qRT-PCR. In E–H, significance was assessed via one-way ANOVA with Dunnett’s post-test comparing all groups to control. Significance calculated on transformed values for H. Significance in comparison to control NPs is represented and denoted: *p < 0.05, **p < 0.01, ***p < 0.001. Error bars are SEM.

Due to the lack of standard immunocompetent mouse models of MCC, to investigate the impact of adding IFNγ to reprogramming NPs *in vivo*, we used the B16F10 mouse model of subcutaneous melanoma to investigate our hypothesis in the most common, systemically aggressive skin cancer type. Given the common usage of ICIs in the clinic, we also sought to explore how our reprogramming NPs would work in combination with anti-PD1. Control, 4-1BBL/IL-12, or 4-1BBL/IL-12/IFNγ NPs were administered intratumorally with or without systemic anti-PD1 antibody (Figure 6A). In vivo transfection of subcutaneous B16F10 melanoma tumors was confirmed by intratumoral administration of fLuc NPs and assessment of luminescence by IVIS (Figure 6B). On day 15, tumors treated with 4-1BBL/IL-12 NPs and anti-PD1 were significantly smaller than those administered control NPs (adjusted *p* = 0.0435) (Figure 6C). Survival based on humane end points was tracked, with a 1.7- and 1.2-fold increase in median survival with 4-1BBL/IL-12 or 4-1BBL/IL-12/IFNγ NPs with anti-PD1 compared to control, indicating significantly improved survival with either treatment (adjusted *p* = 0.0005 and 0.03, respectively) (Figure 6D), while anti-PD1 alone had no significant effect on tumor growth and mouse survival (Figure 6C,D). Interestingly, while some mice treated with 4-1BBL/IL-12/IFNγ NPs and anti-PD1 survived substantially longer than the controls, overall survival in this group was less significant than that of mice treated with 4-1BBL/IL-12 NPs and anti-PD1. Of note, one out of eight animals treated with control NPs and four out of eight animals treated with control NPs with anti-PD1 were euthanized due to signs of distress before the tumor reached 200 mm^2^. Similarly, two out of 15 animals treated with 4-1BBL/IL-12/IFNγ NPs with or without anti-PD1 were euthanized early for similar reasons, whereas no animals were sacrificed prematurely after being treated with 4-1BBL/IL-12 NPs with or without anti-PD1.

### Analysis of Reprogrammed Mouse Melanoma Tumors

Flow cytometry and qPCR 20 days after tumor implantation demonstrated increased immune cell infiltration in tumors receiving 4-1BBL/IL-12/IFNγ NPs when combined with anti-PD1 (Figures 6E, S7, S8B). 4-1BBL/IL12/IFNγ NPs and anti-PD1 drove not just more total immune cells but specifically more T cells, which are predominantly CD8+ (Figures 6F, S8A,B), into the TIME. No substantial difference in NK-cell infiltration was measured with therapeutic particles or anti-PD1 (Figure S8A,B). MHC-I expression on tumor cells was significantly higher in groups treated with 4-1BBL/IL-12 NPs with or without anti-PD1 as well as 4-1BBL/IL-12/IFNγ NPs with or without anti-PD1 (Figure 6G). While qPCR indicated significant IFNγ expression in tumors treated with 4-1BBL/IL-12 NPs and anti-PD1, indicating robust immune activation even in the absence of IFNγ plasmid delivery, IFNγ gene expression was further increased by the addition of the IFNγ plasmid into the NP formulation, with or without anti-PD1 (Figure 6H).

Histologic evaluation showed that tumors receiving 4-1BBL/IL-12 NPs with anti-PD1 or 4-1BBL/IL-12/IFNγ NPs with anti-PD1 were smaller than control tumors and also had increased peritumoral and intratumoral inflammation, demonstrating penetration of TILs into the tumor bulk, and rare cases showed complete immune-mediated tumor regression (Figure S9). Immunohistochemistry (IHC) demonstrated few or no CD8+ T cells (pink) in tumors that received control NPs with or without anti-PD1, but tumors receiving both anti-PD1 and 4-1BBL/IL-12 NPs or 4-1BBL/IL-12/IFNγ NPs revealed significant CD8+ T-cell infiltration distributed peritumorally and throughout the tumor bulk (Figure S9A). Blinded scoring of inflammation and CD8+ T-cell infiltration in these groups indicated increases in both metrics with therapeutic NPs and anti-PD1 compared to control NPs (Figure S9B).

### Analysis of Exhaustion and Polyfunctionality in Reprogrammed Mouse Melanoma Tumors

**Figure 7:**
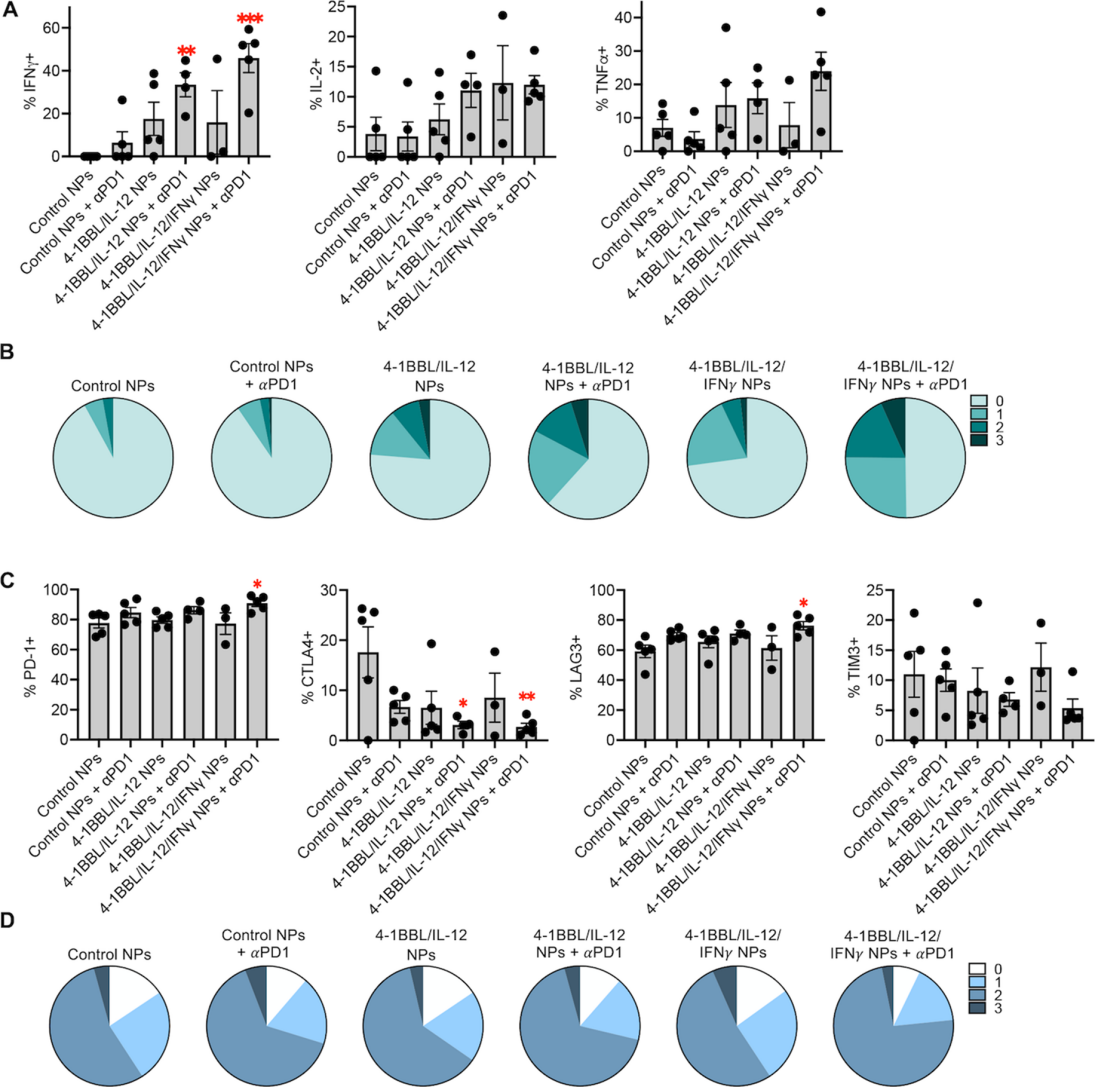
CD8 polyfunctionality and exhaustion analysis in treated B16F10 tumors. B16F10 melanoma tumors (*n* = 5) were treated with intratumoral injections of control (fLuc) NPs, 4-1BBL/IL-12 NPs, or 4-1BBL/IL-12/IFNγ NPs, each with and without intraperitoneal anti-PD1, and then harvested on day 20. Significance in comparison to control NPs was assessed via one-way ANOVA with Dunnett’s post-test and is represented: *p < 0.05, **p < 0.01, ***p < 0.001. Error bars are SEM. (A) Expression of IFNγ, IL-2, and TNFα in CD8+ T cells following stimulation. (B) Functional CD8+ T cell profiles from harvested tumors with stimulation. Each segment represents the proportion of CD8+ T cells expressing 0, 1, 2, or 3 cytokines (IFNγ, IL-2, TNFα). (C) Expression of PD-1, CTLA4, LAG3, and TIM3 on CD8+ T cells from harvested tumors. (D) Exhausted CD8+ T-cell profiles from harvested tumors. Each segment represents the proportion of CD8+ T cells expressing 0, 1, 2, or 3 surface markers (PD-1, LAG3, TIM3).

To examine the mechanism underlying these antitumor immune responses, B16F10 tumors in C57BL/6J mice were dosed as described and then excised and analyzed on day 20. Notably, in this study, two out of five animals treated with 4-1BBL/IL-12/IFNγ NPs were euthanized early due to distressed states. Intracellular cytokine staining (ICS) for IFNγ, IL-2, and TNFα in dissociated tumor cells showed a significantly higher proportion of CD8+ T cells producing IFNγ in tumors treated with 4-1BBL/IL-12 NPs with anti-PD1 or 4-1BBL/IL-12/IFNγ NPs with anti-PD1 compared to control NPs (adjusted *p* = 0.0098 and 0.0003, respectively) (Figures 7A, S10A). Although there were differences in IL-2 and TNFα production that followed similar trends, the results were not statistically significant compared to the control (Figure 7A). Analysis of polyfunctionality, examining the proportion of infiltrating CD8+ T cells secreting 0, 1, 2, or 3 cytokines, showed the highest proportion of T cells producing 1, 2, or 3 cytokines in tumors treated with 4-1BBL/IL-12/IFNγ NPs with anti-PD1. Notably, improved polyfunctionality over the control was also seen with 4-1BBL/IL-12 NPs, which was enhanced by anti-PD-1 (Figure 7B).

Flow cytometry analysis of T-cell exhaustion revealed significantly higher PD1 and LAG3 expression on CD8+ T cells infiltrating tumors treated with 4-1BBL/IL-12/IFNγ NPs and anti-PD1 compared to the control (Figures 7C, S10B). Analysis of the proportion of CD8+ T cells expressing 0, 1, 2, or 3 exhaustion markers (PD1, LAG3, and TIM3) showed that NPs including the IFNγ plasmid, in the absence of anti-PD1 antibody, led to a higher proportion of terminally exhausted cells expressing three exhaustion markers than 4-1BBL/IL-12 NPs only (Figure 7D). Analysis of CD4+ T cells showed that, without anti-PD1, tumors treated with 4-1BBL/IL-12/IFNγ NPs had a greater proportion of T cells expressing 2 or more exhaustion markers (Figure S11A,B).

## Discussion

Here we describe biodegradable PBAE NPs delivering plasmids that encode immunostimulatory molecules to reprogram the TIME after local administration. These reprogramming NPs can modulate MHC expression and elicit a targeted immune response in MCC *in vitro* and in melanoma *in vivo*. *In vitro* coculture bioassays with MCC cells showed that CD8+ T cells could kill significant numbers of tumor cells even without any additional treatment when cocultured with the MCC26 cell line, which had the highest baseline level of MHC-I expression (Figure S5B). This was not seen with MCC13 and UISO, which both have low baseline MHC-I expression, and thus transfection with immunostimulatory genes was required for CD8+ T-cell killing of these tumor cells. This suggested that a treatment that increases MHC-I expression in MCC cells could lead to more efficient T cell-mediated tumor cell killing.

Interferons restore MHC-I expression *ex vivo* in MCC cells with low baseline expression,^12^ and we observed that downstream IFNγ production in early studies correlated with increased MHC-I expression (Figure 3). We therefore incorporated IFNγ DNA into NP formulations to modulate the MHC-I expression in MCC cells and melanoma tumors. As our therapeutic approach relies on antigen presentation for antigen-specific T-cell activation, we hypothesized that adding an additional factor to drive antigen presentation would potentiate an early effector antitumor immune response. *In vitro* transfection with 4-1BBL/IL-12/IFNγ NPs led to robust increases in MHC-I and MHC-II expression due to local production of IFNγ by the tumor cells themselves (Figure 5A,B,D). In the clinic, low-dose IFNγ has been tested to increase MHC-I expression and generate a “hot” TIME, with modest results.^20,21^ Toxicity with systemic IFNγ can be dose-related, however,^22^ and we anticipated that local production of IFNγ would maximize response and dose tolerability while minimizing toxicity.

When combined with immune cells *in vitro* and in a mouse model of melanoma *in vivo*, local supplementation of IFNγ via codelivery was not required for effective induction of MHC expression and strong antitumor immunity. Coculture of MCC cells with CD8+ T cells or PBMCs demonstrated increased IFNγ production, T-cell expansion, and targeted cancer cell killing with 4-1BBL/IL-12 NPs compared to controls (Figures 3A,B, S6), with greater differences seen in MCC13 and UISO compared to MCC26 due to lower baseline T cell-mediated killing. This suggests that the formulation with only 4-1BBL and IL-12 was sufficient to elicit an immune response in an *ex vivo* system, inducing sufficient endogenous IFNγ production from cocultured or infiltrating T cells to drive MHC expression, adaptive immune recognition, and antitumor immune responses.

Analysis of treated tumors revealed significantly higher MHC-I expression on tumor cells with both 4-1BBL/IL-12 NPs and 4-1BBL/IL-12/IFNγ NPs as well as 4-1BBL/IL-12 NPs with anti-PD1 *in vivo*, and there was a trend toward higher MHC-I expression in the group treated with 4-1BBL/IL-12/IFNγ NPs and anti-PD-1. *In vivo* treatment with 4-1BBL/IL-12 NPs with anti-PD1 and 4-1BBL/IL-12/IFNγ NPs with and without anti-PD1 significantly increased the level of IFNγ expression in the tumor (Figure 6H). Intriguingly, despite increased levels of IFNγ production seen with IFNγ plasmid-containing NP groups, these groups were not the most effective at increasing survival (Figure 6D). This could be explained by differences in T-cell function in addition to T-cell numbers as well as systemic effects from IFNγ reaching systemic circulation. When we evaluated T-cell functionality, ICS showed that CD8+ T cells in tumors treated with 4-1BBL/IL-12/IFNγ NPs and anti-PD1 were substantially more polyfunctional than in the control and nominally more than in mice treated with NPs without IFNγ (Figure 7B). However, importantly, the group with IFNγ did show the most exhausted phenotype with higher levels of exhaustion markers, suggesting that T-cell exhaustion may have contributed to the reduced effectiveness of NPs carrying all three plasmids (Figure 7D). Interestingly, the NK-cell response was not as prominent *in vivo* as *in vitro* with MCC, indicating that the response *in vivo* was predominantly T-cell driven (Figure S8A,B).

Notably, in multiple studies, the mice administered 4-1BBL/IL-12/IFNγ NPs showed an increased likelihood of premature death compared to 4-1BBL/IL-12 alone (4 mice euthanized in total vs 1 in total, respectively, across the studies), although some of the longest survivors were in the group that included IFNγ. Dissection of these animals revealed blood in the stomach, suggestive of an excessive immune response leading to colitis, an adverse event sometimes caused by ICI therapy.^23^ This large variability, along with the death of some mice due to adverse immune events, suggests that, while IFNγ drives improved MHC expression and may be partially beneficial, leading to a robust antitumor response in some mice, local IFNγ may have a narrow therapeutic index. *In vivo* data show several orders of magnitude increased IFNγ expression from codelivery of the IFNγ plasmid, irrespective of anti-PD1 administration, suggesting both the potential for systemic toxicity from systemic leak as well as the potential of overshooting the potential therapeutic window from local delivery of IFNγ (Figure 6H).

This NP-based gene-delivery system has potential not only for elucidating immunological mechanisms but also as a translational cancer therapy. Clinical trials on IL-12 gene therapy for metastatic MCC and melanoma demonstrated that local gene therapy can cause tumor regression.^24−27^ In trials utilizing skin electroporation as the method of gene delivery, patients reported site pain as an adverse effect, although these trials on local plasmid delivery demonstrate the clinical feasibility of using local cytokine gene delivery to treat cancer. The use of nanoparticles to enhance gene delivery in patients has seen a surge in regulatory approval as well as general public acceptance due to recent mRNA vaccine technologies,^28^ although lipid nanoparticles (LNPs) are associated with rare but potentially severe adverse events.^29^ Although LNPs have also been used for mRNA delivery to tumors for immunotherapy,^30,31^ the use of plasmid DNA/polymeric NPs in the current work promotes greater flexibility in the design and stability of the cargo, enables cost-effective manufacturing of the polymeric NPs, entails a fully degradable vehicle as a carrier to attenuate toxicity concerns, and allowed us to modularly and thoroughly explore the mechanism of action of each genetic component of the immunotherapy.

## Conclusions

In this study, we investigate the mechanisms underlying genetic reprogramming of skin cancer using PBAE NPs to deliver plasmids for signal 2 and 3 molecules. We hypothesized that reprogramming NPs would restore MHC-I expression in cells with transcriptionally repressed MHC-I, bypassing a major resistance mechanism, and lead to immune activation against cancer cells. Overexpression of 4-1BBL and IL-12 alone was sufficient to activate T cells and drive MHC-I expression *in vitro* if immune cells were present in coculture. Local supplementation of IFNγ drove MHC-I and MHC-II expression *in vitro*, restoring the local antigen presentation that was otherwise downregulated by the cancer cells. *In vivo*, 4-1BBL/IL-12 NPs with anti-PD1 were sufficient to activate T cells, leading to IFNγ production, MHC-I/II induction, smaller tumors, and prolonged survival. Local delivery of IFNγ, which significantly increased IFNγ expression in the TIME compared to 4-1BBL/IL-12 NPs, resulted in improved CD8+ T-cell polyfunctionality but potentially also led to a more exhausted phenotype and systemic toxicity in some cases. This modular approach of PBAE NP-mediated TIME reprogramming has significant potential for the treatment of MCC and melanoma.

## Supporting Information Available

PBAE monomer structures and synthesis; cellular viability screening of PBAEs in MCC cell lines; polymer and nanoparticle characterization; flow cytometry gating strategies for coculture assays; optimization of MCC PBMC coculture assay; results of coculture of human MCC cells with human PBMCs; flow cytometry gating strategy for ex vivo tumor analysis; flow cytometry and qPCR analysis of reprogrammed mouse melanoma tumors; histology of reprogrammed mouse melanoma tumors; flow cytometry gating strategies for polyfunctionality and exhaustion analysis; and exhaustion analysis on CD4+ cells from treated B16F10 tumors (PDF).

## References

[ref1] Hughes M. P.; Hardee M. E.; Cornelius L. A.; Hutchins L. F.; Becker J. C.; Gao L. Merkel Cell Carcinoma: Epidemiology, Target, and Therapy. Curr. Dermatol. Rep. 2014, 3 (1), 46–53. 10.1007/s13671-014-0068-z. 24587977 PMC3931972

[ref2] Schadendorf D.; Fisher D. E.; Garbe C.; Gershenwald J. E.; Grob J. J.; Halpern A.; Herlyn M.; Marchetti M. A.; McArthur G.; Ribas A.; Roesch A.; Hauschild A. Melanoma. Nat. Rev. Dis. Prim. 2015, 1, 15003 10.1038/nrdp.2015.3. 27188223

[ref3] D’Angelo S. P.; Bhatia S.; Brohl A. S.; Hamid O.; Mehnert J. M.; Terheyden P.; Shih K. C.; Brownell I.; Lebbé C.; Lewis K. D.; Linette G. P.; Milella M.; Georges S.; Shah P.; Ellers-Lenz B.; Bajars M.; Güzel G.; Nghiem P. T. Avelumab in Patients with Previously Treated Metastatic Merkel Cell Carcinoma: Long-Term Data and Biomarker Analyses from the Single-Arm Phase 2 JAVELIN Merkel 200 Trial. J. Immunother. Cancer 2020, 8 (1), e000674 10.1136/jitc-2020-000674. 32414862 PMC7239697

[ref4] Nghiem P.; Bhatia S.; Lipson E. J.; Sharfman W. H.; Kudchadkar R. R.; Brohl A. S.; Friedlander P. A.; Daud A.; Kluger H. M.; Reddy S. A.; Boulmay B. C.; Riker A. I.; Burgess M. A.; Hanks B. A.; Olencki T.; Margolin K.; Lundgren L. M.; Soni A.; Ramchurren N.; Church C.; Park S. Y.; Shinohara M. M.; Salim B.; Taube J. M.; Bird S. R.; Ibrahim N.; Fling S. P.; Moreno B. H.; Sharon E.; Cheever M. A.; Topalian S. L. Durable Tumor Regression and Overall Survival in Patients with Advanced Merkel Cell Carcinoma Receiving Pembrolizumab as First-Line Therapy. J. Clin. Oncol. 2019, 37 (9), 693–702. 10.1200/JCO.18.01896. 30726175 PMC6424137

[ref5] Munhoz R. R.; Postow M. A. Clinical Development of PD-1 in Advanced Melanoma. Cancer J. 2018, 24 (1), 7–14. 10.1097/PPO.0000000000000299. 29360722 PMC5819364

[ref6] Nghiem P. T.; Bhatia S.; Lipson E. J.; Kudchadkar R. R.; Miller N. J.; Annamalai L.; Berry S.; Chartash E. K.; Daud A.; Fling S. P.; Friedlander P. A.; Kluger H. M.; Kohrt H. E.; Lundgren L.; Margolin K.; Mitchell A.; Olencki T.; Pardoll D. M.; Reddy S. A.; Shantha E. M.; Sharfman W. H.; Sharon E.; Shemanski L. R.; Shinohara M. M.; Sunshine J. C.; Taube J. M.; Thompson J. A.; Townson S. M.; Yearley J. H.; Topalian S. L.; Cheever M. A. PD-1 Blockade with Pembrolizumab in Advanced Merkel-Cell Carcinoma. N. Engl. J. Med. 2016, 374 (26), 2542–2552. 10.1056/NEJMoa1603702. 27093365 PMC4927341

[ref7] Mellman I.; Coukos G.; Dranoff G. Cancer Immunotherapy Comes of Age. Nature 2011, 480 (7378), 480–489. 10.1038/nature10673. 22193102 PMC3967235

[ref8] Smyth M. J.; Godfrey D. I.; Trapani J. A. A Fresh Look at Tumor Immunosurveillance and Immunotherapy. Nat. Immunol. 2001, 2 (4), 293–299. 10.1038/86297. 11276199

[ref9] Berry S.; Giraldo N. A.; Green B. F.; Cottrell T. R.; Stein J. E.; Engle E. L.; Xu H.; Ogurtsova A.; Roberts C.; Wang D.; Nguyen P.; Zhu Q.; Soto-Diaz S.; Loyola J.; Sander I. B.; Wong P. F.; Jessel S.; Doyle J.; Signer D.; Wilton R.; Roskes J. S.; Eminizer M.; Park S.; Sunshine J. C.; Jaffee E. M.; Baras A.; De Marzo A. M.; Topalian S. L.; Kluger H.; Cope L.; Lipson E. J.; Danilova L.; Anders R. A.; Rimm D. L.; Pardoll D. M.; Szalay A. S.; Taube J. M. Analysis of Multispectral Imaging with the AstroPath Platform Informs Efficacy of PD-1 Blockade. Science 2021, 372 (6547), eaba2609 10.1126/science.aba2609. 34112666 PMC8709533

[ref10] Paulson K. G.; Voillet V.; McAfee M. S.; Hunter D. S.; Wagener F. D.; Perdicchio M.; Valente W. J.; Koelle S. J.; Church C. D.; Vandeven N.; Thomas H.; Colunga A. G.; Iyer J. G.; Yee C.; Kulikauskas R.; Koelle D. M.; Pierce R. H.; Bielas J. H.; Greenberg P. D.; Bhatia S.; Gottardo R.; Nghiem P.; Chapuis A. G. Acquired Cancer Resistance to Combination Immunotherapy from Transcriptional Loss of Class I HLA. Nat. Commun. 2018, 9 (1), 3868 10.1038/s41467-018-06300-3. 30250229 PMC6155241

[ref11] Dhatchinamoorthy K.; Colbert J. D.; Rock K. L. Cancer Immune Evasion Through Loss of MHC Class I Antigen Presentation. Front. Immunol. 2021, 12, 636568 10.3389/fimmu.2021.636568. 33767702 PMC7986854

[ref12] Paulson K. G.; Tegeder A.; Willmes C.; Iyer J. G.; Afanasiev O. K.; Schrama D.; Koba S.; Thibodeau R.; Nagase K.; Simonson W. T.; Seo A.; Koelle D. M.; Madeleine M.; Bhatia S.; Nakajima H.; Sano S.; Hardwick J. S.; Disis M. L.; Cleary M. A.; Becker J. C.; Nghiem P. Downregulation of MHC-I Expression Is Prevalent but Reversible in Merkel Cell Carcinoma. Cancer Immunol. Res. 2014, 2 (11), 1071–1079. 10.1158/2326-6066.CIR-14-0005. 25116754 PMC4221542

[ref13] Wang D.; Tai P. W. L.; Gao G. Adeno-Associated Virus Vector as a Platform for Gene Therapy Delivery. Nat. Rev. Drug Discov. 2019, 18 (5), 358–378. 10.1038/s41573-019-0012-9. 30710128 PMC6927556

[ref14] Bhise N. S.; Shmueli R. B.; Gonzalez J.; Green J. J. A Novel Assay for Quantifying the Number of Plasmids Encapsulated by Polymer Nanoparticles. Small 2012, 8 (3), 367–373. 10.1002/smll.201101718. 22139973 PMC3838880

[ref15] Shmueli R. B.; Bhise N. S.; Green J. J. Evaluation of Polymeric Gene Delivery Nanoparticles by Nanoparticle Tracking Analysis and High-Throughput Flow Cytometry. J. Vis. Exp. 2013, 73, e50176 10.3791/50176. PMC3622088 23486314

[ref16] Tzeng S. Y.; Green J. J. Subtle Changes to Polymer Structure and Degradation Mechanism Enable Highly Effective Nanoparticles for SiRNA and DNA Delivery to Human Brain Cancer. Adv. Healthc. Mater. 2013, 2 (3), 468–480. 10.1002/adhm.201200257. 23184674 PMC3838886

[ref17] Tzeng S. Y.; Patel K. K.; Wilson D. R.; Meyer R. A.; Rhodes K. R.; Green J. J. In Situ Genetic Engineering of Tumors for Long-Lasting and Systemic Immunotherapy. Proc. Natl. Acad. Sci. U. S. A. 2020, 117 (8), 4043–4052. 10.1073/pnas.1916039117. 32034097 PMC7049107

[ref18] Zhou F. Molecular Mechanisms of IFN-γ to Up-Regulate MHC Class I Antigen Processing and Presentation. Int. Rev. Immunol. 2009, 28 (3–4), 239–260. 10.1080/08830180902978120. 19811323

[ref19] Zhang S.; Kohli K.; Black R. G.; Yao L.; Spadinger S. M.; He Q.; Pillarisetty V. G.; Cranmer L. D.; Van Tine B. A.; Yee C.; Pierce R. H.; Riddell S. R.; Jones R. L.; Pollack S. M. Systemic Interferon-γ Increases MHC Class I Expression and T-Cell Infiltration in Cold Tumors: Results of a Phase 0 Clinical Trial. Cancer Immunol. Res. 2019, 7 (8), 1237–1243. 10.1158/2326-6066.CIR-18-0940. 31171504 PMC6677581

[ref20] Propper D. J.; Chao D.; Braybrooke J. P.; Bahl P.; Thavasu P.; Balkwill F.; Turley H.; Dobbs N.; Gatter K.; Talbot D. C.; Harris A. L.; Ganesan T. S. Low-Dose IFN-γ Induces Tumor MHC Expression in Metastatic Malignant Melanoma. Clin. Cancer Res. 2003, 9 (1), 84–92. 12538455

[ref21] Zhang S.; Kohli K.; Graeme Black R.; Yao L.; Spadinger S. M.; He Q.; Pillarisetty V. G.; Cranmer L. D.; Van Tine B. A.; Yee C.; Pierce R. H.; Riddell S. R.; Jones R. L.; Pollack S. M. Systemic Interferon-g Increases MHC Class I Expression and T-Cell Infiltration in Cold Tumors: Results of a Phase 0 Clinical Trial. Cancer Immunol. Res. 2019, 7 (8), 1237–1243. 10.1158/2326-6066.CIR-18-0940. 31171504 PMC6677581

[ref22] Sriskandan K.; Garner P.; Watkinson J.; Pettingale K. W.; Brinkley D.; Calman F. M. B.; Tee D. E. H. A Toxicity Study of Recombinant Interferon-Gamma given by Intravenous Infusion to Patients with Advanced Cancer. Cancer Chemother. Pharmacol. 1986, 18 (1), 63–68. 10.1007/BF00253067. 3093108

[ref23] Puzanov I.; Diab A.; Abdallah K.; Bingham C. O.; Brogdon C.; Dadu R.; Hamad L.; Kim S.; Lacouture M. E.; LeBoeuf N. R.; Lenihan D.; Onofrei C.; Shannon V.; Sharma R.; Silk A. W.; Skondra D.; Suarez-Almazor M. E.; Wang Y.; Wiley K.; Kaufman H. L.; Ernstoff M. S. Managing Toxicities Associated with Immune Checkpoint Inhibitors: Consensus Recommendations from the Society for Immunotherapy of Cancer (SITC) Toxicity Management Working Group. J. Immunother. Cancer 2017, 5 (1), 95 10.1186/s40425-017-0300-z. 29162153 PMC5697162

[ref24] Mahvi D. M.; Henry M. B.; Albertini M. R.; Weber S.; Meredith K.; Schalch H.; Rakhmilevich A.; Hank J.; Sondel P. Intratumoral Injection of IL-12 Plasmid DNA – Results of a Phase I/IB Clinical Trial. Cancer Gene Ther. 2007, 14 (8), 717–723. 10.1038/sj.cgt.7701064. 17557109

[ref25] Bhatia S.; Longino N. V.; Miller N. J.; Kulikauskas R.; Iyer J. G.; Ibrani D.; Blom A.; Byrd D. R.; Parvathaneni U.; Twitty C. G.; Campbell J. S.; Le M. H.; Gargosky S.; Pierce R. H.; Heller R.; Daud A. I.; Nghiem P. Intratumoral Delivery of Plasmid IL12 via Electroporation Leads to Regression of Injected and Noninjected Tumors in Merkel Cell Carcinoma. Clin. Cancer Res. 2020, 26 (3), 598–607. 10.1158/1078-0432.CCR-19-0972. 31582519 PMC9868004

[ref26] Daud A. I.; DeConti R. C.; Andrews S.; Urbas P.; Riker A. I.; Sondak V. K.; Munster P. N.; Sullivan D. M.; Ugen K. E.; Messina J. L.; Heller R. Phase I Trial of Interleukin-12 Plasmid Electroporation in Patients with Metastatic Melanoma. J. Clin. Oncol. 2008, 26 (36), 5896–5903. 10.1200/JCO.2007.15.6794. 19029422 PMC2645111

[ref27] Algazi A. P.; Tsai K. K.; Rosenblum M.; Fox B. A.; Andtbacka R. H. I.; Li A.; Takamura K. T.; Dwyer M.; Browning E.; Talia R.; Twitty C.; Le M. H.; Gargosky S.; Campbell J. S.; Ballesteros-Merino C.; Bifulco C. B.; Pierce R.; Daud A. Immune Monitoring Outcomes of Patients with Stage III/IV Melanoma Treated with a Combination of Pembrolizumab and Intratumoral Plasmid Interleukin 12 (PIL-12). J. Clin. Oncol. 2017, 35 (7_suppl), 78 10.1200/JCO.2017.35.7_suppl.78.28034075

[ref28] Verbeke R.; Lentacker I.; De Smedt S. C.; Dewitte H. The Dawn of MRNA Vaccines: The COVID-19 Case. J. Control. Release 2021, 333, 511–520. 10.1016/j.jconrel.2021.03.043. 33798667 PMC8008785

[ref29] Meo S. A.; Bukhari I. A.; Akram J.; Meo A. S.; Klonoff D. C. COVID-19 Vaccines: Comparison of Biological, Pharmacological Characteristics and Adverse Effects of Pfizer/BioNTech and Moderna Vaccines. Eur. Rev. Med. Pharmacol. Sci. 2021, 25 (3), 1663–1679. 10.26355/eurrev_202102_24877. 33629336

[ref30] Liu J. Q.; Zhang C.; Zhang X.; Yan J.; Zeng C.; Talebian F.; Lynch K.; Zhao W.; Hou X.; Du S.; Kang D. D.; Deng B.; McComb D. W.; Bai X. F.; Dong Y. Intratumoral Delivery of IL-12 and IL-27 MRNA Using Lipid Nanoparticles for Cancer Immunotherapy. J. Control. Release 2022, 345, 306–313. 10.1016/j.jconrel.2022.03.021. 35301053 PMC9133152

[ref31] Hewitt S. L.; Bai A.; Bailey D.; Ichikawa K.; Zielinski J.; Karp R.; Apte A.; Arnold K.; Zacharek S. J.; Iliou M. S.; Bhatt K.; Garnaas M.; Musenge F.; Davis A.; Khatwani N.; Su S. V.; MacLean G.; Farlow S. J.; Burke K.; Frederick J. P. Durable Anticancer Immunity from Intratumoral Administration of IL-23, IL-36γ, and OX40L MRNAs. Sci. Transl. Med. 2019, 11 (477), eaat9143 10.1126/scitranslmed.aat9143. 30700577

