# Correction to “Biomaterial-Mediated Genetic Reprogramming of Merkel Cell Carcinoma and Melanoma Leads to Targeted Cancer Cell Killing *In Vitro* and *In Vivo*”

**DOI:** 10.1021/acsbiomaterials.4c02308

**Published:** 2024-12-16

**Authors:** Kathryn M. Luly, Jordan J. Green, Joel C. Sunshine, Stephany Y. Tzeng

The following inadvertent error appears in this article: for all *in vivo* tumor studies, the polymer-to-DNA ratio in our nanoparticles is listed as 30:1 (30 w/w). The correct ratio is 36 w/w for all *in vivo* studies. The conclusions of the manuscript are not at all affected by this error. We sincerely apologize for the inconvenience to readers and editors.

