# Supplementary material for: Biomaterial-Mediated Genetic Reprogramming of Merkel Cell Carcinoma and Melanoma Leads to Targeted Cancer Cell Killing In Vitro and In Vivo
Source: ACS Biomater Sci Eng. 2023 Oct 5;9(11):6438–50. doi: 10.1021/acsbiomaterials.3c00885 (PMC10646862; doi:10.1021/acsbiomaterials.3c00885)
Supplement: Supplementary file 1 — ab3c00885_si_001.pdf [file ab3c00885_si_001.pdf]

Supplemental Information

Biomaterial-Mediated Genetic Reprogramming of Merkel Cell Carcinoma and Melanoma Leads to Targeted Cancer Cell Killing *In Vitro* and *In Vivo*

Kathryn M Luly[1,2], Jordan J Green[1,2,3,4,5,6,7], Joel C Sunshine[1,8]*, Stephany Y Tzeng[1,2]*

[1]Department of Biomedical Engineering, Johns Hopkins University, Baltimore, MD, 21205, USA; [2]Translational Tissue Engineering Center, Johns Hopkins University School of Medicine, Baltimore, MD, 21231, USA; [3]Institute for Nanobiotechnology, Johns Hopkins University, Baltimore, MD, 21218, USA; [4]Bloomberg~Kimmel Institute for Cancer Immunotherapy, Johns Hopkins University School of Medicine, Baltimore, MD, 21231, USA; [5]Sidney Kimmel Comprehensive Cancer Center, Johns Hopkins University School of Medicine, Baltimore, MD, 21231, USA; [6]Departments of Neurosurgery, Ophthalmology, and Oncology, Johns Hopkins University School of Medicine, Baltimore, MD, 21231, USA; [7]Departments of Materials Science & Engineering and Chemical & Biomolecular Engineering, Johns Hopkins University, Baltimore, MD, 21218, USA; [8]Departments of Dermatology and Pathology, Johns Hopkins University School of Medicine, Baltimore, MD, 21287, USA

*Correspondence should be addressed to:

Joel C Sunshine
Department of Dermatology, Pathology, and Biomedical Engineering, Johns Hopkins University, Baltimore, MD, USA
600 N. Wolfe Street, Blalock 907
Baltimore, MD 21231
joelsunshine@jhmi.edu
Phone: 410-955-5933
Fax: 443-287-6298

Stephany Y Tzeng
Department of Biomedical Engineering, Johns Hopkins University, Baltimore, MD, USA
400 N Broadway, Smith 5039
Baltimore, MD 21231
stzeng1@jhmi.edu
Phone: 443-287-5536
Fax: 443-287-6298

Keywords
Nanomaterials; Immunotherapy; Gene delivery; Melanoma; Merkel Cell Carcinoma

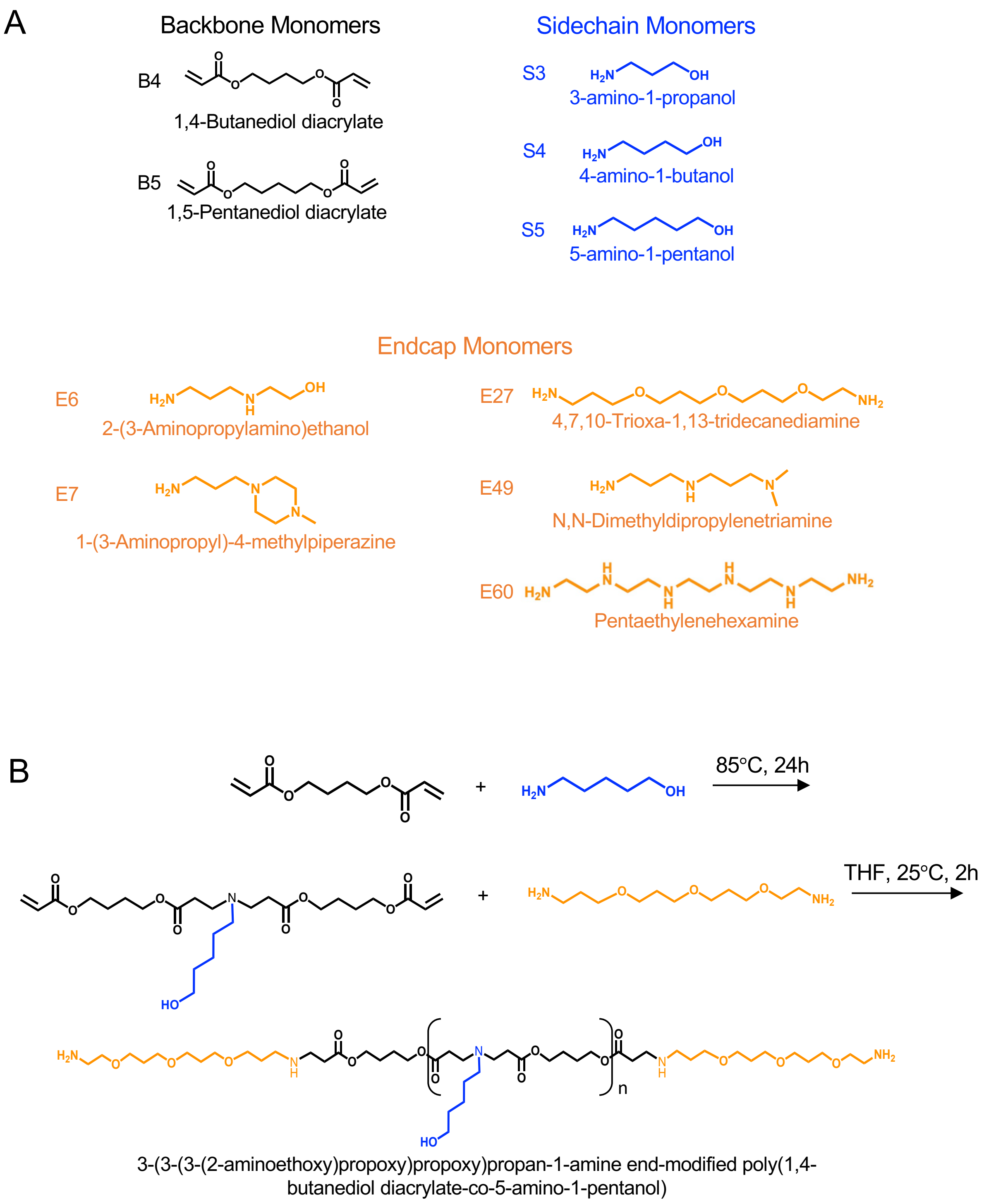

Figure S1. PBAE monomer structures and synthesis. (A) Structures of backbone, B, sidechain, S, and endcap, E, monomers used to synthesize PBAE polymers in this study. (B) Synthetic route of model PBAE 4-5-27.

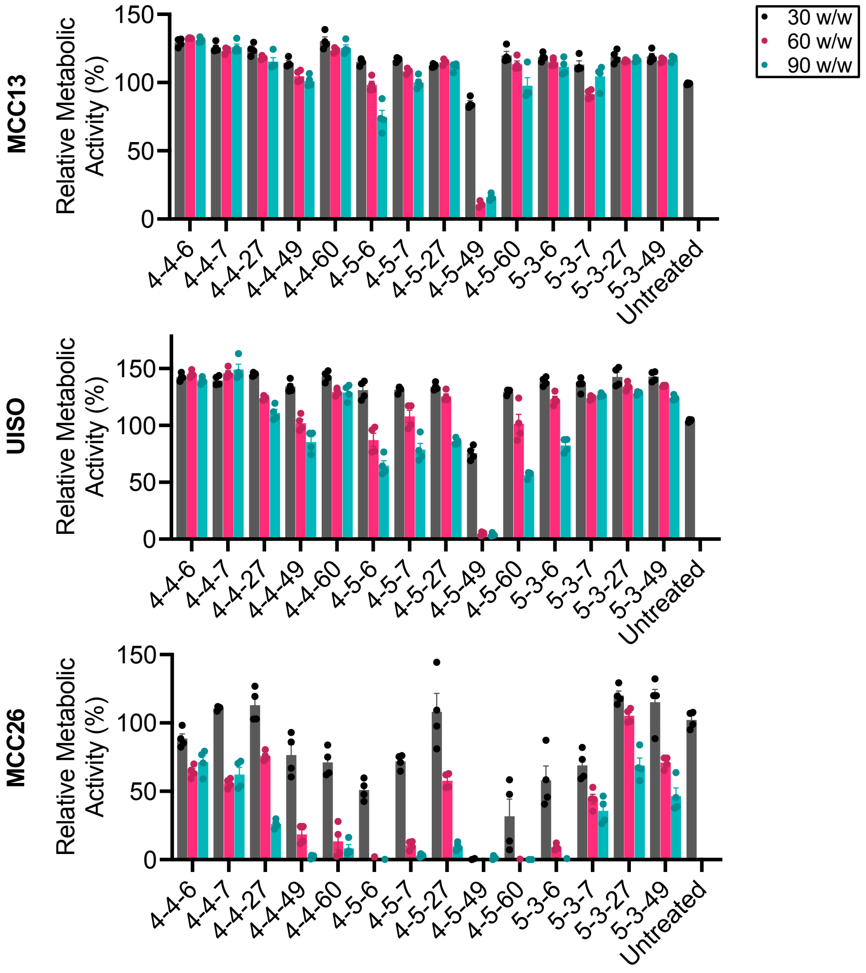

Figure S2. Cellular viability screening of PBAEs in MCC cell lines. PBAE NPs delivered 600 ng GFP DNA at 30, 60, and 90 w/w. Relative metabolic activity, as a measure of cellular viability, was assessed via MTS assay 24 h following transfection.

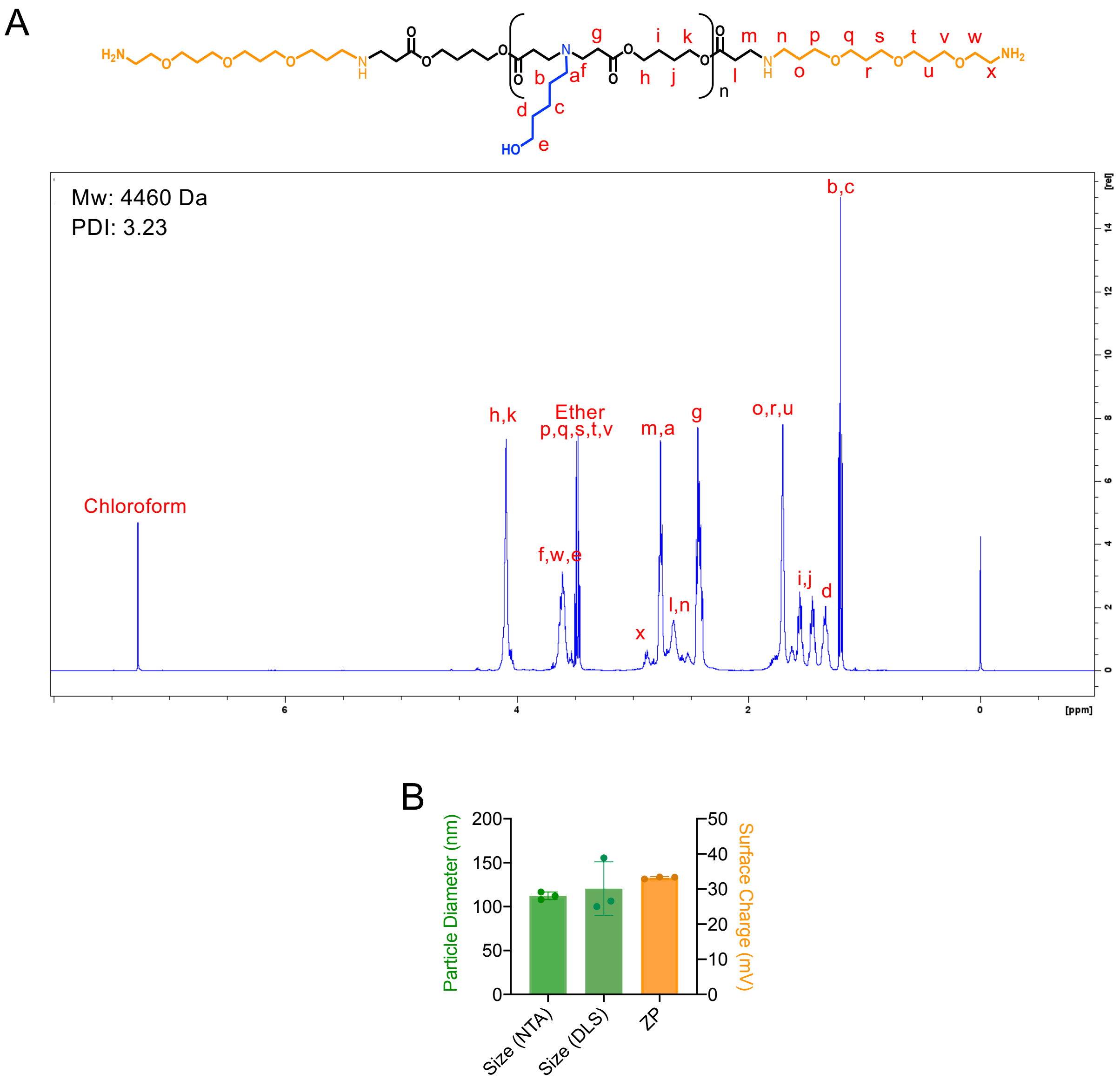

Figure S3. Polymer and NP characterization. (A) To fully characterize the chosen PBAE, 4-5-27, we performed NMR and GPC to assess the structure and determine the molecular weight, respectively. NMR analysis confirmed the structure of 4-5-27 (Fig. S5A), and GPC indicated a number average molecular weight of 4460 Da and a polydispersity of 3.23. (B) Particle sizing was performed on the resulting NPs via dynamic light scattering (DLS) and nanoparticle-tracking analysis (NTA). NPs had a mean particle hydrodynamic diameter of 112 nm with a standard deviation of 4.4 nm by number-averaged NTA and 120 nm with a standard deviation of 30.3 nm as the intensity-weighted Z-average by DLS. The zeta potential was +33.2 mV with a standard deviation of 0.3 mV. Replicate measurements were conducted using individually prepared NP samples.

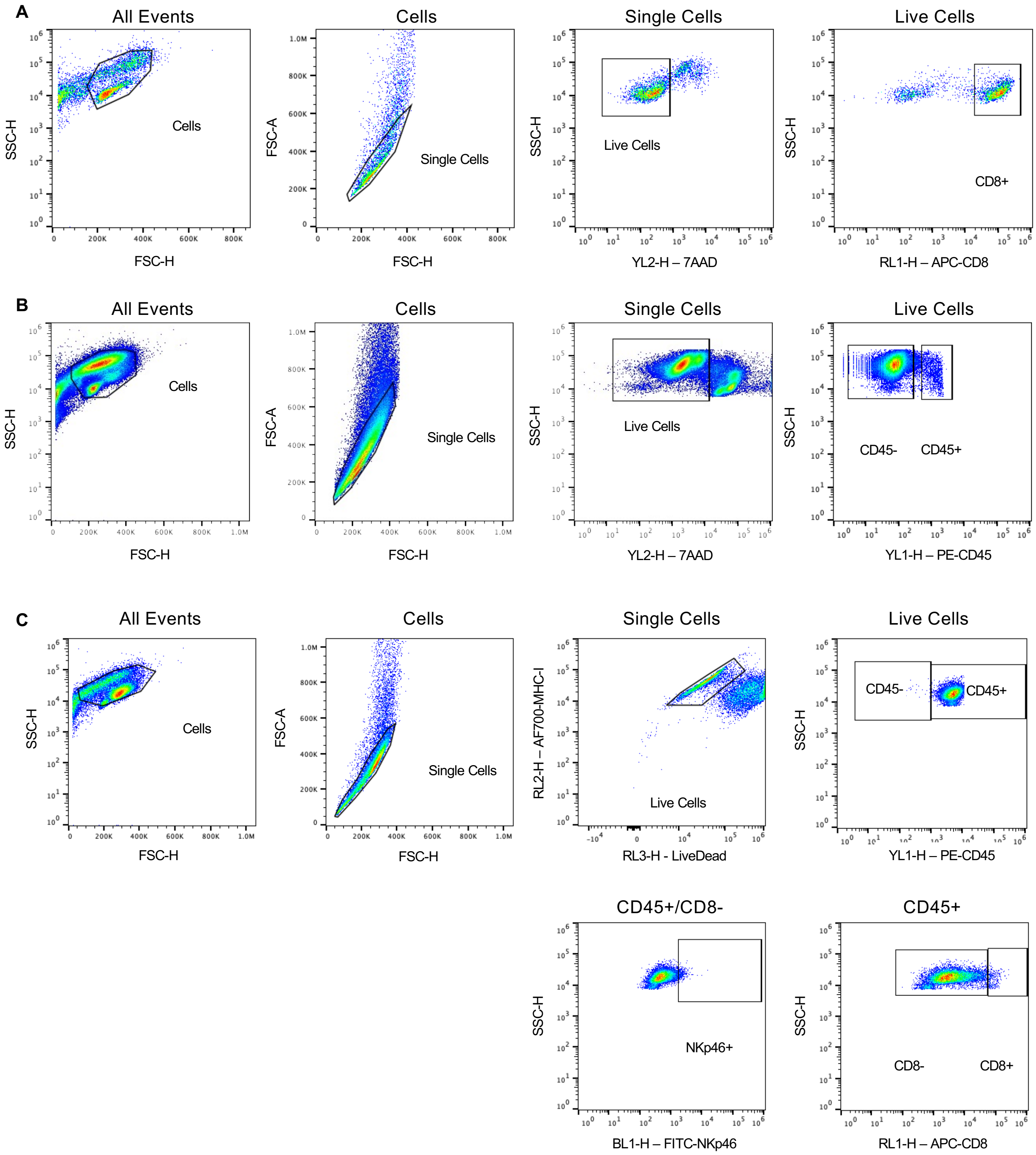

Figure S4. Flow cytometry gating strategies. (A-B) Gating strategy for the CD8+ T cell co-culture assay. (C) Gating strategy for PBMC co-culture assay.

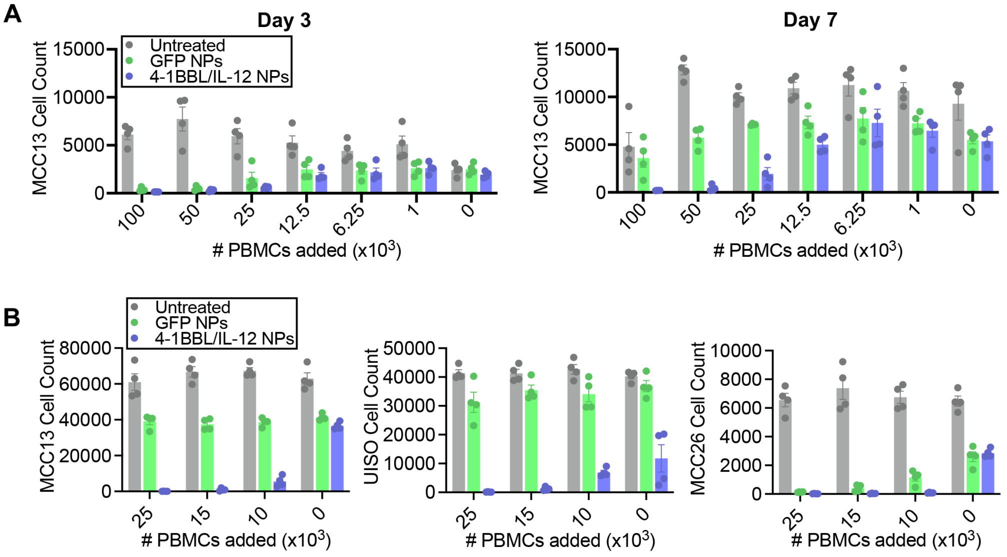

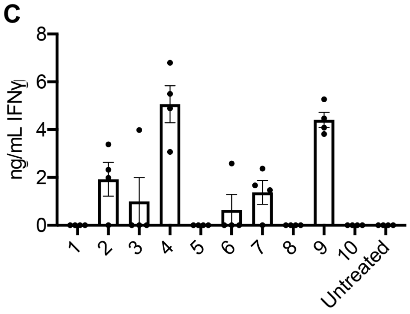

Condition	ng 4-1BBL	ng IL-12	ng IFNγ	ng GFP	Total ng DNA
1	112.5	337.5			450
2	112.5	337.5	100		550
3	112.5	337.5		100	550
4	112.5	337.5	200		650
5	112.5	337.5		200	650
6	112.5	437.5			550
7	112.5	437.5	100		650
8	112.5	437.5		100	650
9	112.5	437.5	200		750
10	112.5	437.5		200	750

Figure S5. Optimization of MCC PBMC co-culture assay. (A) Human PBMCs were added to MCC13 cells that were either untreated or transfected with GFP or 4-1BBL/IL-12 NPs. MCC13 cell count was assessed on days 3 and 7. (B) Human PBMCs were added to MCC13, UISO, or MCC26 cells that were either untreated or transfected with GFP or 4-1BBL/IL-12 NPs. MCC cell count was assessed on day 7. (C) Formulation optimization to incorporate an IFNγ plasmid demonstrated robust secretion in MCC13 48 h following transfection.

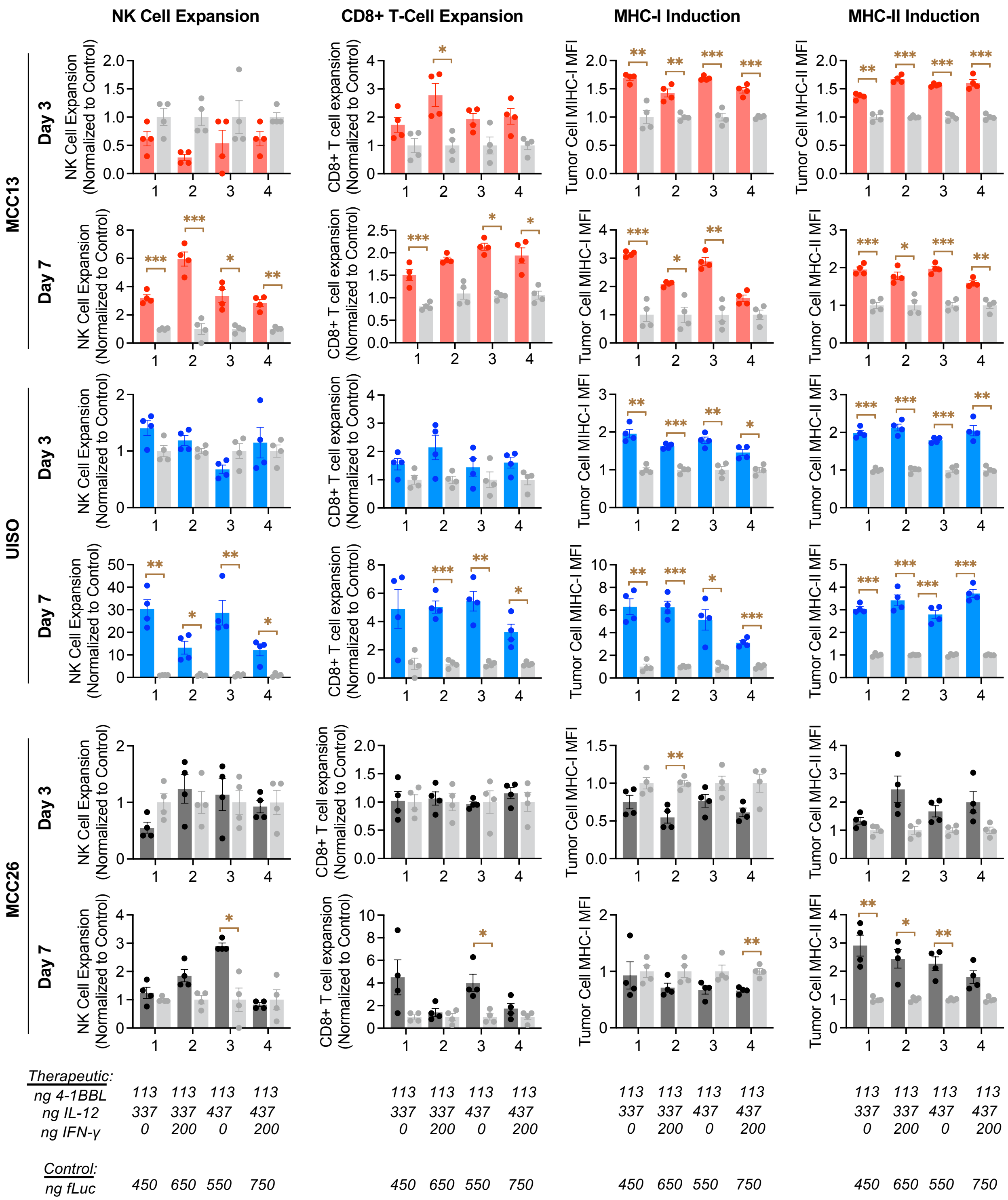

Figure S6. Co-culture of human MCC cells with human PBMCs. MCC cells were transfected with NPs delivering control plasmid (fLuc) or plasmids for 4-1BBL and IL-12 with and without IFNγ (ng amounts of each plasmid are indicated). Human PBMCs were added 24h following transfection. NK-cell expansion, T-cell expansion, MHC-I induction, and MHC-II induction were assessed compared to matched controls via flow cytometry 3 and 7 days following the addition of PBMCs. Significance, via one-way ANOVA comparing each group to its matched control with Bonferroni correction for multiple comparisons, for each cell line is represented. Significance is denoted: *, p<0.05; **,p<0.01; ***, p<0.001. Error bars are SEM.

A

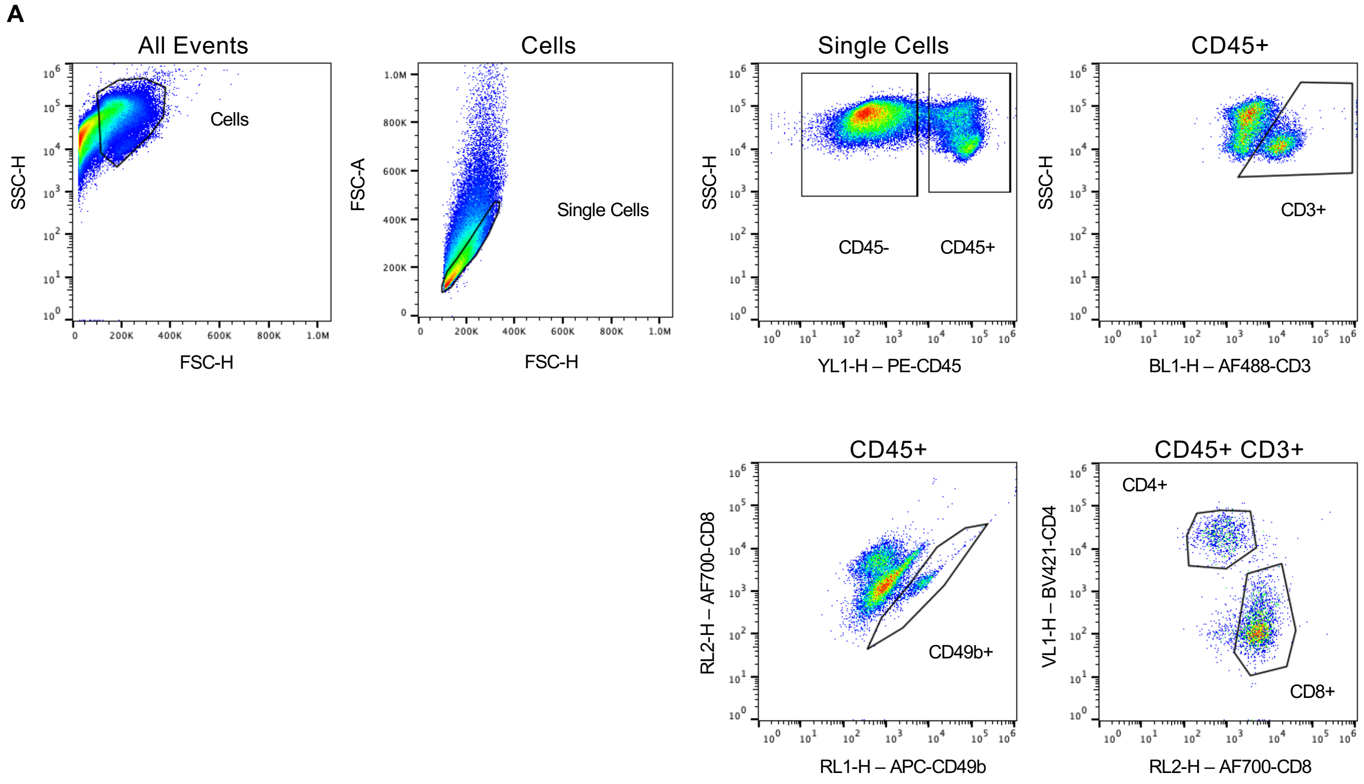

Figure S7. Gating strategy for *ex vivo* tumor analysis.

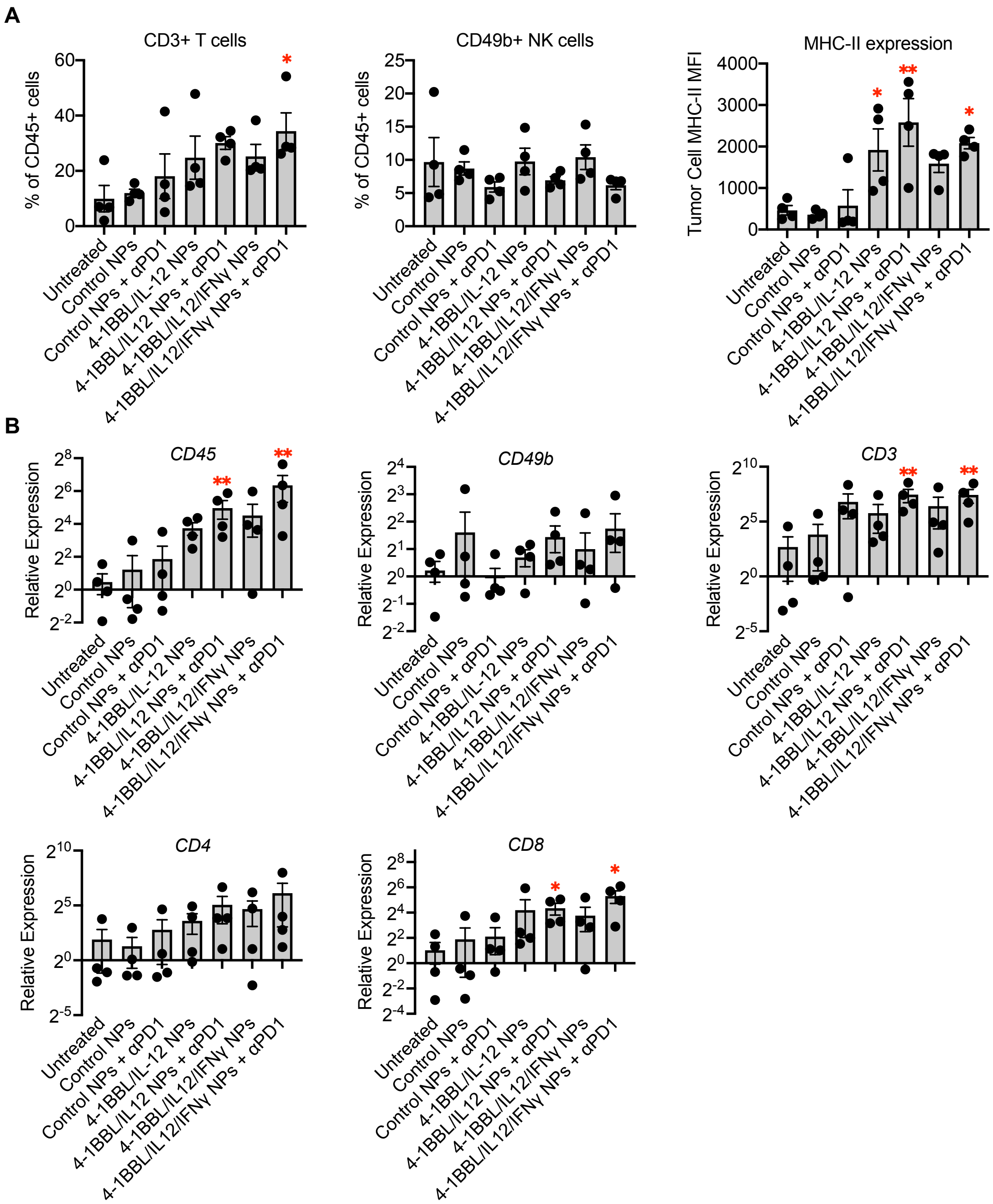

Figure S8. Analysis of reprogrammed mouse melanoma tumors. B16F10 melanoma tumors were treated with intratumoral injections of control (fLuc) NPs, 4-1BBL/IL-12 NPs, or 4-1BBL/IL-12/IFNγ NPs, each with and without intraperitoneal αPD1 (n=4 per group). Tumors were harvested on day 20. Error bars are SEM. (A) Assessment of CD3+ T-cell infiltration and NK-cell infiltration via flow cytometry. (B) CD45, CD49b, CD3, CD4, and CD8 gene expression in tumors assessed via qRT-PCR. Significance was assessed via one-way ANOVA with Dunnett's post-test comparing all groups to control, and was calculated on transformed values. Significance in comparison to control NPs is denoted: *p<0.05, **p<0.01, ***p<0.001. Error bars are SEM.

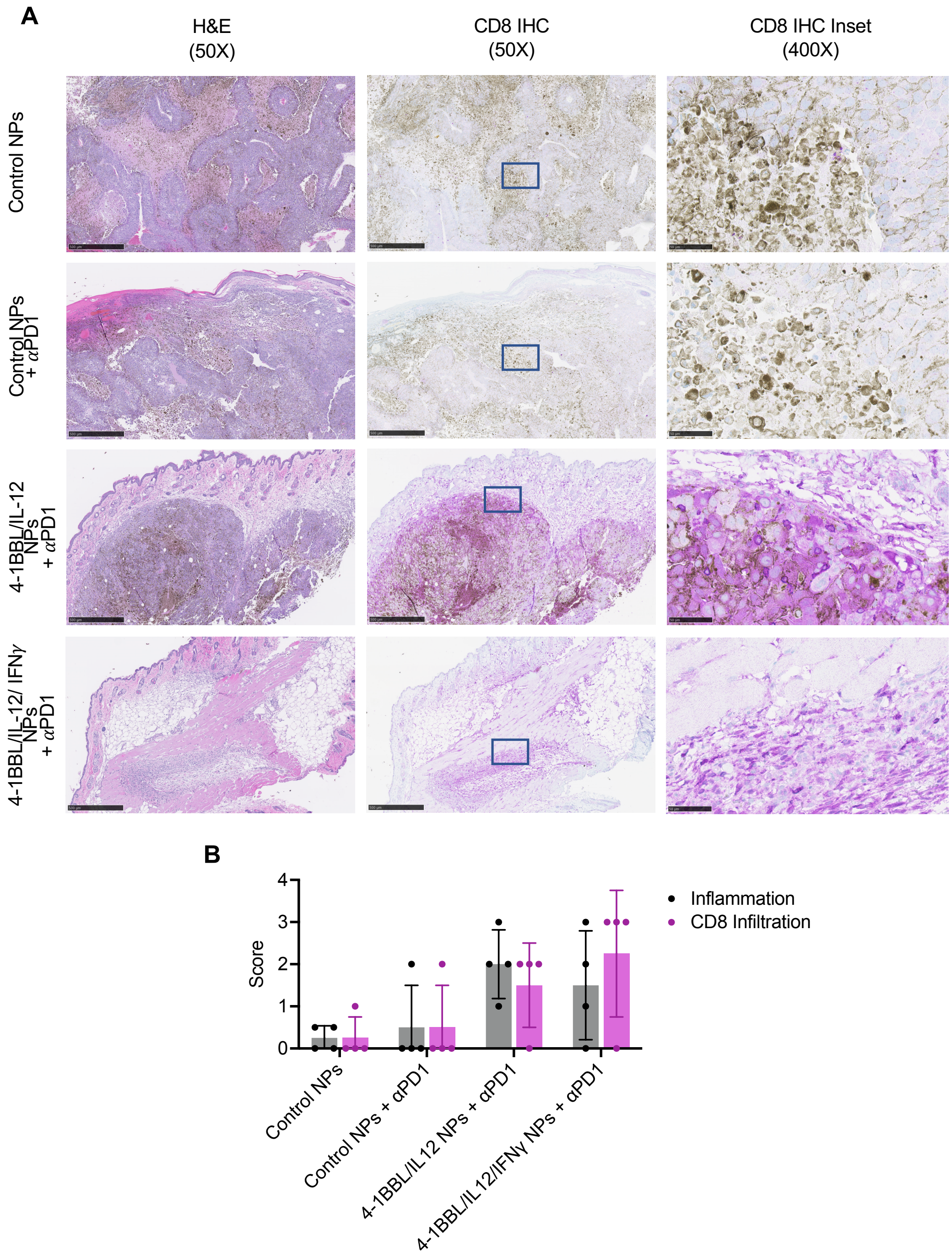

Figure S9. Histology of reprogrammed mouse melanoma tumors. B16F10 melanoma tumors (n=4) were treated with intratumoral injections of control (fLuc) NPs, 4-1BBL/IL-12 NPs, or 4-1BBL/IL-12/IFNγ NPs, each with and without intraperitoneal anti-PD1. Tumors were harvested on day 20. (A) Tumors were sectioned and stained with H&E and CD8 (pink). Representative section images shown. Images are 50X. Scalebars are 500 μm. CD8 insets are 400X and scalebars are 50 μm. (B) Semi-quantitative, blinded scoring of H&E- and CD8-stained slides was performed (0=no inflammation/CD8 infiltration, 1=mild inflammation/CD8 infiltration, 2=moderate inflammation/CD8 infiltration, 3=strong inflammation/CD8 infiltration).

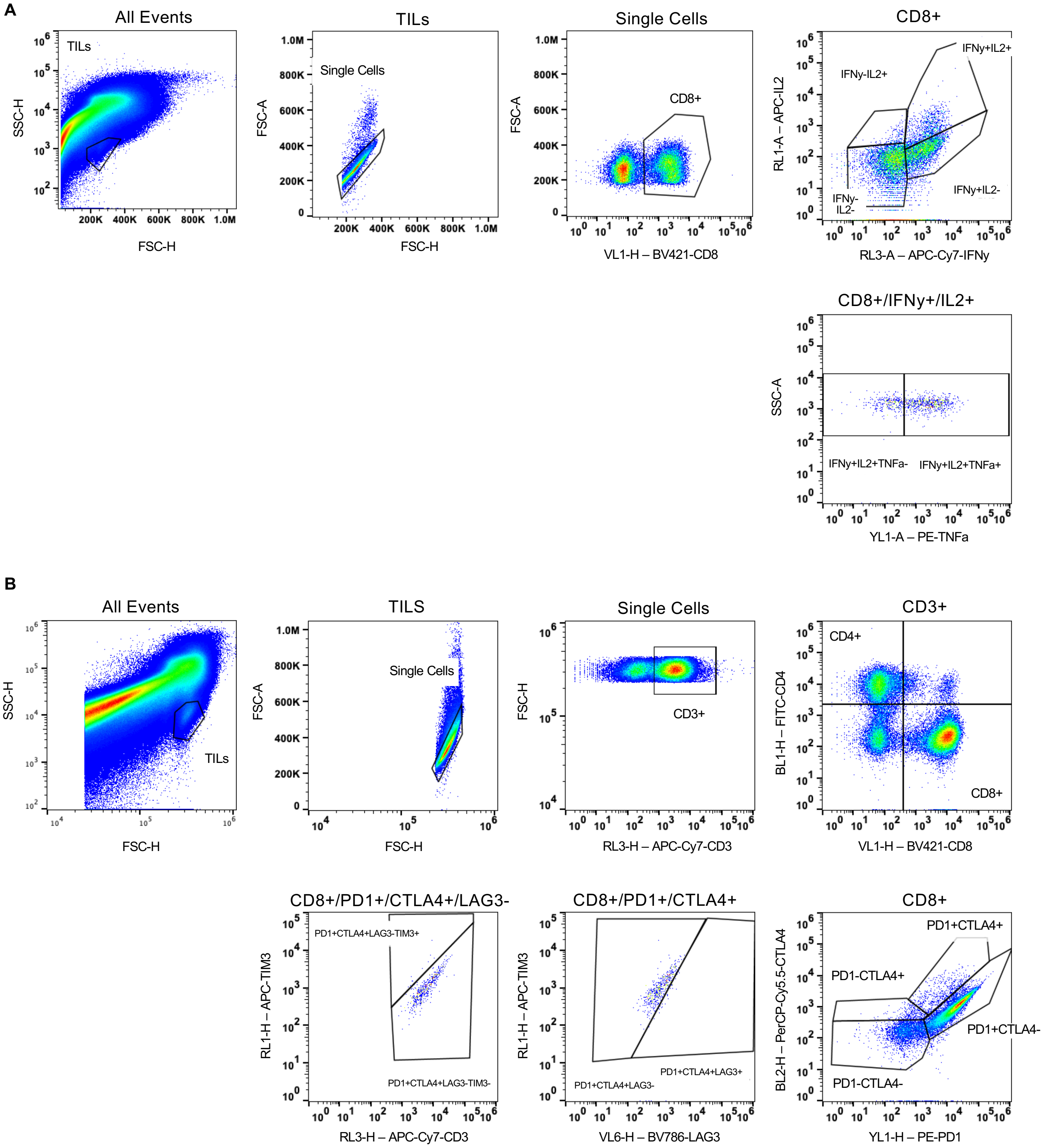

Figure S10. Flow cytometry gating strategies. (A) Gating strategy for CD8+ T-cell polyfunctionality analysis. (B) Gating strategy for CD8+ T-cell exhaustion analysis.

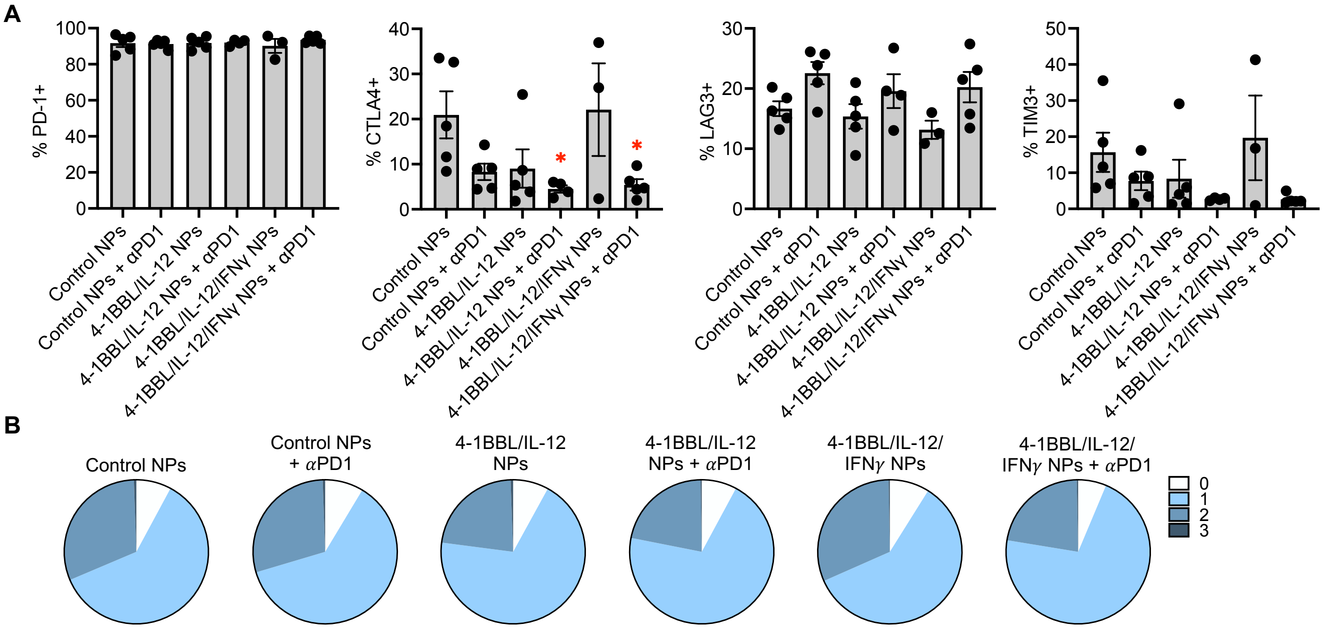

Figure S11. Exhaustion markers in treated B16F10 tumors. B16F10 melanoma tumors (n=5) were treated with intratumoral injections of control (fLuc) NPs, 4-1BBL/IL-12 NPs, or 4-1BBL/IL-12/IFNγ NPs, each with and without intraperitoneal anti-PD1. Tumors were harvested on day 20. (A) Expression of PD-1, CTLA4, LAG3, and TIM3 on CD4+ T cells from harvested tumors. (B) Exhausted CD4+ T-cell profiles from harvested tumors. Each segment represents the proportion of CD4+ T cells expressing 0, 1, 2, or 3 surface markers (PD-1, LAG3, TIM3). Significance in comparison to control NPs was assessed via one-way ANOVA with Dunnett's post-test and is represented: *p<0.05, **p<0.01, ***p<0.001. Error bars are SEM.

Table S1. Antibodies used for staining in CD8+ co-culture assay

Target	Fluorophore	Clone	Manufacturer	Cat.No.	Dilution
CD45	PE	HI30	Biolegend	304058	666
MHC I	AF700	W6/32	Biolegend	311438	40
CD8	APC	SK1	Biolegend	344722	40

Table S2. Human-reactive antibodies used for staining in PBMC co-culture assay

Target	Fluorophore	Clone	Manufacturer	Cat.No.	Dilution
CD45	PE	HI30	Biolegend	304058	333
MHC I	AF700	W6/32	Biolegend	311438	20
MHC II	PerCP/Cy5.5	Tü39	Biolegend	361710	20
CD8	APC	SK1	Biolegend	344722	20
NKp46	FITC	9E2	Biolegend	331922	20
CD4	PerCP/Cy5.5	SK3	Biolegend	344608	20

Table S3. Mouse-reactive antibodies used for staining in *ex vivo* tumor analysis

Target	Fluorophore	Clone	Manufacturer	Cat.No.	Dilution
CD8	AF700	53-6.7	Biolegend	100730	200
CD3	AF488	17A2	Biolegend	100210	100
CD49b	APC	DX5	Biolegend	108910	80
CD45	PE	30-F11	Biolegend	103106	100
CD4	BV421	RM4-5	Biolegend	100563	100
IA/IE	AF700	M5/114.15.2	Biolegend	107622	100
H2Kb/H2Db	PerCP/Cy5.5	28-8-6	Biolegend	114620	40

Table S4. Mouse-reactive antibodies used for staining in *ex vivo* tumor exhaustion analysis

Target	Fluorophore	Clone	Manufacturer	Cat.No.	Dilution
CD8	BV421	53-6.7	Biolegend	100737, 100753	20
LAG-3	BV785	C9B7W	Biolegend	125219	40
CD4	FITC	RM4-5	Biolegend	100510	200
CTLA4	PerCP/Cy5.5	UC10-4B9	Biolegend	106316	20
PD-1	PE	29F.1A12	Biolegend	135206	20
TIM-3	APC	B8.2C12	Biolegend	134008	80
CD3	APC-Cy7	17A2	Biolegend	100222	80

Table S5. Mouse-reactive antibodies used for staining in *ex vivo* ICS

Target	Fluorophore	Clone	Manufacturer	Cat.No.	Dilution
TNFa	PE	MP6-XT22	Biolegend	506306	80
IL-2	APC	JES6-5H4	Biolegend	503810	80
IFNy	APC/Cy7	XMG1.2	Biolegend	505849	200
CD8	BV421	53-6.7	Biolegend	100737, 100753	20
